# CpG Island Methylation of Suppressor of Cytokine Signaling-1 Gene Induced by HCV Is Associated With HCV-Related Hepatocellular Carcinoma

**DOI:** 10.3389/fmicb.2022.679593

**Published:** 2022-06-06

**Authors:** Miao Liu, Lingyao Du, Xing Cheng, Man Yuan, Jin Shang, Ying Shi, Hailing Yang, Hong Tang

**Affiliations:** ^1^Center of Infectious Diseases, West China Hospital of Sichuan University, Chengdu, China; ^2^Department of Hepatobiliary-Pancreatic Surgery, Cell Transplantation Center, Sichuan Provincial People’s Hospital, University of Electronic Science and Technology of China, Chengdu, China; ^3^School of Medicine, University of Electronic Science and Technology of China, Chengdu, China; ^4^Molecular Oncology Research Institute, Tufts Medical Center, Boston, MA, United States; ^5^Graduate Program in Cellular and Molecular Physiology, School of Graduate Biomedical Sciences, Tufts University, Boston, MA, United States

**Keywords:** CpG island, methylation, *SOCS-1*, HCV, hepatocellular carcinoma

## Abstract

Suppressor of cytokine signaling 1 (SOCS-1) is implicated in both virus infection and carcinogenesis. This study investigated the role of HCV infection on SOCS-1 in normal and HCV-infected tissues and revealed a possible mechanism underlying HCV-induced hepatocellular carcinoma (HCC) genesis. In total, 10 HCV-HCC tissues, seven adjacent tissues, seven distal tissues, and 16 normal liver tissues were collected. SOCS-1 expression in tissue sections was detected by immunohistochemistry. After viral load was quantified, the correlation between SOCS-1 expression and viral load was analyzed in different tissues. Then, HCV replicon model was used to detect a relationship between HCV and SOCS-1. Subsequently, methylation-specific PCR (MSP) was applied to show the methylation status of *SOCS-1* genes in normal tissues and HCV-replicating cell lines. A correlation between gene methylation, SOCS-1 expression, and HCV was analyzed. The lowest expression of SOCS-1 was observed in HCV-HCC tissues. Tissues with a higher HCV viral load showed lower SOCS-1 expression (*p* = 0.0282). Consistently, *SOCS-1* mRNA and protein were lower in HCV-replicating cell lines than in uninfected ones. Furthermore, gene methylation was found in all examined tissues but higher in HCC tissues, and it is positively correlated with HCV viral load (*r*^2^ = 0.7309, *p* < 0.0001). HCV infection would upregulate methylation of the *SOCS-1* gene in HCV-replicating cell lines. The downregulation of SOCS-1 in normal and HCV-replicating cell lines may result from HCV infection through epigenetic regulation, in which gene methylation in the CpG island of *SOCS-1* promoters upon HCV infection suppresses its expression.

## Introduction

Globally, there are more than 100 million patients with chronic HCV infection, while more than 10 million patients are found in China (Rao et al., 2014). HCV infection is characterized by chronicity, symptomless progression, and tumorigenicity. Medical screening is the only effective way to distinguish patients in the early stage. Without proper medical intervention, a quarter of patients with chronic hepatitis C (CHC) would deteriorate and end up with cirrhosis, liver failure, or HCC (Chen and Morgan, 2006; Corey et al., 2010). The significant development of direct-acting antivirals (DAAs) for HCV has improved the disease prognosis; more than 90% of patients can be cured by DAAs. However, disease progression will not stop even after the virus is eliminated in some patients, especially the ones with decompensated cirrhosis (Du and Tang, 2016). As many patients are diagnosed at the end stage of liver diseases, a large disease population is still facing the risk of HCC even with access to DAAs. Therefore, clarifying the mechanism of HCV-induced HCC genesis can help HCV infected patients in better managing both virus infection and virus-induced diseases.

When HCV infects liver cells, JAK/STAT and other inflammatory signaling pathways are activated and contribute to the elimination of the virus (Stone et al., 2013). But overactivation of inflammatory signaling pathways can result in the overproduction of reactive oxygen species and affect the stability of telomeres and genomes, increasing the risk of HCC (Waris and Ahsan, 2006; Medvedev et al., 2016). Negative regulators, including suppressor of cytokine signaling 1 (SOCS-1), can act on those pathways to suppress the overactivation (O’sullivan et al., 2007). SOCS-1 is also an antitumor factor that could directly regulate oncogenes and cell proliferation (Rottapel et al., 2002). SOCS-1 was downregulated in the multiple types of cancers due to hypermethylation of the CpG island in its gene promoter (Galm et al., 2003; Qu et al., 2013; Wang et al., 2015; Kang et al., 2016).

The expression of SOCS-1 in HCV-HCC remains unknown. There are only a few studies focusing on the role SOCS-1 plays between HCV and HCC. Since SOCS-1 is related to both viral infection and carcinogenesis, SOCS-1 may be involved in HCV-induced hepatocarcinogenesis. This study aimed to assess the expression of SOCS-1 in HCV-HCC tissues and the correlation between HCV infection and SOCS-1 expression in normal and verified HCV replicons. How HCV regulated *SOCS-1* CpG hypermethylation of *SOCS-1* promoters was assessed to examine its correlation with HCV infection. In sum, we show a possible mechanism underlying HCV-induced HCC induction.

## Materials and Methods

### Study Materials

The HCV replicon JFH-1 was kindly given as a gift by Tetsuro Shimakami, Kanazawa University, under the permission of Apath. L. L., United States. The HCV replicative cell line Huh7.5.1 was kindly given as a gift by Limin Chen, Institution of Blood Transfusion, Chinese Academy of Medical Sciences.

Serum samples were obtained from CHC outpatients consulted at the Hepatology Clinic, West China Hospital of Sichuan University. The diagnosis was made according to WHO guidelines for the screening, care, and treatment of patients with chronic hepatitis C, April 2016. The baseline serum before treatment was collected.

Tissue samples of HCV-HCC were obtained from the pathologic specimen bank of West China Hospital, Sichuan University. Each set of tissues contains cancer tissue, adjacent tissue, and distal tissue. We used the electronically recorded diagnosis “HCC” through international classification of diseases, tenth revision (ICD-10), and positive serum anti-HCV or HCV RNA as an index to identify these tissues.

Normal liver tissues were obtained from the specimen bank in the Department of Forensic Pathology, West China School of Basic and Forensic Medicine, Sichuan University.

### Detection of HCV Core Protein and Suppressor of Cytokine Signaling-1 in Tissues

We applied immunohistochemistry (IHC) to detect HCV particles and cytokines in tissues and cells. Histological sections were prepared. Mouse anti-HCV core protein monoclonal antibody (sc-58144, Santa Cruz, CA, United States) and Rabbit anti-SOCS-1 polyclonal antibody (sc-9021, Santa Cruz, CA, United States) were used as primary antibodies. The HRP Polymer anti-Rabbit/Mouse broad-specificity antibody (Gene, China) was used as the secondary antibody. After being stained with 3′,3′-diaminobenzidine tetrahydrochloride (DAB) and counterstained with hematoxylin, the sections were mounted and evaluated. Positive staining of HCV particles and SOCS-1 presented as particles in the hepatocytes. The percentage of positive hepatocytes and their staining intensity were evaluated. Axiotis scoring criteria were applied. The percentage score ranged from 0 to 4, representing 0–10, 11–25, 26–50, 51–75, and 76–100%, respectively. The intensity score ranged from 0 to 3, representing no color, yellow, brown, and tan. The sum of the percentage score and the intensity score equaled the sum score. Five different sum scores from a random high-power field (400×) were obtained for a mean sum score. The assessment was implemented by two pathologists unaware of the tissue section arrangement. If there is a difference in their opinions, the average of their scores will be used as the final score (Du et al., 2014).

### Assessment of Histological Inflammation in Non-HCC Tissues

We applied Knodell scoring, also known as histology activity index (HAI), in grading and staging the histological inflammation. After staining with hematoxylin and eosin, the periportal ± bridging necrosis (piecemeal necrosis), intralobular degeneration and focal necrosis, and portal inflammation were scored according to the criteria in the “original form of histology activity index” (Goodman, 2007). Necroinflammatory scores were obtained after these three scores were totaled. Similarly, five different necroinflammatory scores from a random high-power field (400×) were obtained for a mean score, and assessments from two pathologists unaware of the tissue section arrangement were followed.

### Detection and Quantification of HCV RNA in Tissues

Tissues were grinded into powder in liquid nitrogen and lysed in Trizol LS reagents (Invitrogen, Life Technologies, Carlsbad, CA, United States). Total RNAs were extracted according to users’ instructions. Then, they were reversely transcribed into cDNA with an RNA reverse transcription kit (PrimeScript™ RT reagent Kit with gDNA Eraser, Takara Bio Inc., Dalian, China). HCV RNA was detected through nested real-time PCR (Casanova et al., 2014).

The amplification was performed in two steps. The first round of PCR reaction was performed with the first pair of primers targeting 5′ UTR HCV sequences (pHCV1-Forward (F): 5′-CCCCTGTGAGGAACTWCTGTCTTCACGC-3′; pHCV1-Reverse (R): 5′-AGGTTTAGGATTTGTGCTCAT-3′), and the volume of reaction mixture was 20 μl, consisting of 10 μl template cDNA, 2 μl 10 × buffer (Mg^2+^ free), 2.4 μl MgCl_2_ (25 mM), 2 μl dNTP mixture (2.5 mM each), 1 μl 10pM pHCV1-F, 1 μl 10pM pHCV1-R, 0.2 μl Taq DNA polymerase, and 1.4 μl double distilled H_2_O (Ex Taq Kit, Takara Bio Inc., Dalian, China). The reaction was performed for 15 cycles at 94°C for 30 s, 60°C for 30 s, and 72°C for 30 s in S100PCR (Bio-Rad, Hercules, CA, United States).

The second round of nested real-time PCR was performed with the second pair of primers targeting fragments inside the production from the first reaction (pHCV2-F: 5′-GAAAGCGYCTAGCCATGGCGTTAG-3′; pHCV2-R: 5′-ACGGTCTACGAGACCTCCCGGGGC-3′). A probe labeled with the fluorophore FAM and the quencher TAMRA (5′-CACCCTATCAGGCAGTACCACAAGGCC-3′) was applied. The second amplification was performed in a reaction volume of 30 μl consisting of 1.4 μl production from the first reaction, 3 μl 10 × buffer (Mg^2+^ free), 3.6 μl MgCl_2_ (25 mM), 3 μl dNTP mixture (2.5 mM each), 0.375 μl 10pM pHCV1-F, 0.375 μl 10pM pHCV1-R, 0.125 μl 10pM probe, 0.3 U Taq DNA polymerase, and 17.825 μl double distilled H_2_O. The reaction was performed from predenatured at 95°C for 600 s, followed by 35 cycles at 95°C for 15 s, 60°C for 60 s in Roche LightCycler96 (Roche diagnostic, GmbH).

Through nested real-time PCR, the Cq value of HCV RNA was obtained. A standard curve was needed to calculate the viral load. In this study, we applied a serum sample with a known viral load (10^6^ copies/ml) to establish the standard curve. The viral RNA in the serum sample was extracted and reverse-transcribed into cDNA according to the procedures mentioned above. Notably, 10-fold serial dilutions (10^2^–10^6^ copies/ml) of the cDNA were used as templates in nested real-time PCR. Then, a standard curve was established to quantify HCV RNA (*y* = −3.265*x* + 31.32, *R*^2^ = 0.993).

Then, tissue cells were counted through quantitative real-time PCR of cellular β-actin and estimation of 6.667 pg of β-actin cDNA/cells in our previous study (Liang et al., 2016). The real-time PCR of cellular β-actin was performed in the same reaction system and conditions as the second round of amplification in nested real-time PCR with different primer pairs and probe (β-actin-F: 5′-ACTGTGCCCATCTACGAGG-3′; β-actin-R: 5′-CAGGCAGC TCGTAGCTCTT-3′; β-actin probe: FAM-5′-CGGGAAAT CGTGCGTGAC-3′-TAMRA). Subsequently, the Cq values obtained were converted into a quantitative amount of β-actin cDNA with previously verified standard curve (*y* = −3.16*x* + 40.16, *R*^2^ = 0.995). Then, the viral load in hepatocytes were calculated through the equation “HCV/hepatocytes (copies/cell) = HCV viral load (copies/ml)/β-actin (pg/ml) × 6.667 (pg/cell).”

### Establishment of HCV-Replicating Cell Lines and Detection of Cell-Cultured Viral Particles

HCV strained JFH-1 was cloned from a Japanese patient with fulminant hepatitis. The full genomic replicon was constructed using neomycin-resistant genes, namely, EMCV IRES and JFH-1 cDNA. Replicon RNA was synthesized *in vitro* first and was then transfected into Huh7 cells. These cells were cultured along with G418. The independent colonies screened out were HCV-replicating cell lines. The culture supernatant contained infectious cell-cultured viral particles (HCVcc), which is eligible to infect Huh7.5.1 cells (Zhong et al., 2005; Date et al., 2007). Then, culture supernatant containing HCVcc 1 μg was added into every 2-ml medium in 6-well culture plates with Huh7.5.1 cells. After 3 days, cells were collected for viral detection or other experiments. HCV RNA in cultural supernatant and cells was detected in the same way as that in serum and tissues mentioned before.

### Detection of Suppressor of Cytokine Signaling-1 mRNA and Protein Expression in Cells

We used real-time PCR to detect mRNA level of *SOCS-1*. Collected cells were lysed and mRNA was extracted. A DNase treatment was performed to clear the genomic DNA. Then, they were reversely transcribed into cDNA with an RNA reverse transcription kit (PrimeScript™ RT reagent 21 Kit with gDNA Eraser, Takara Bio Inc., Dalian, China). A pair of primer locating on *SOCS-1* gene (SOCS-1-F: 5′-CACGCACTTCCGC ACATTCC-3′; SOCS-1-R: 5′TCCAGCAGCTCGAAGAGGCA-3′), and another pair of primer locating GAPDH gene (GAPDH-F: 5′-ACCCACTCCTCCACCTTTGA-3′, GAPDH-R: 5′-CTGTTGCTGTAGCCAAATTCGT-3′) were used to amplify *SOCS-1* cDNA and internal reference, respectively. The amplification was implemented with Fast Start Universal SYBR Green Master in LightCycler 96 (Roche diagnostic, GmbH). The Minimum Information for Publication of Quantitative Real-Time PCR Experiment (MIQE) guidelines were followed in all gene expression analyzes. Melting curve analyzes were conducted to check the specificity of the qPCR products. Non-template controls were included in each experiment to detect contaminations. A known concentration sample was included in each experiment to detect and correct potential inter-assay variations. All reactions were performed three times.

We used Western blot to detect SOCS-1 expression in cells. Cells were lysed for total proteins. After quantitation, total proteins were separated by electrophoresis in an SDS polyacrylamide gel and transferred to a PVDF membrane. With the same multifunctional primary antibody as before and HRP-linked goat against rabbit secondary antibody (ZSGBBIO, Beijing, China), SOCS-1 was marked and visualized using a chemiluminescent substrate (ThermoFisher, Waltham, MA, United States) and ChemiDoc_MP imaging system (Bio-Rad, Hercules, CA, United States). The band intensity of SOCS-1 expression was semiquantified in the same imaging system. Immunocytochemistry (ICC) was applied when detecting SOCS-1 expression. Also, the primary antibody of SOCS-1 and its working concentration were the same as those in IHC in tissues.

### Detection of CpG Island Methylation of the Suppressor of Cytokine Signaling-1 Gene in Host Cells

Methylation-specific PCR (MSP) was used to detect the methylation status of the CpG island of the host *SOCS-1* gene. Cells or tissues were lysed in Trizol LS reagents (Invitrogen, Life Technologies, Carlsbad, CA, United States) and genomic DNA was extracted according to users’ instructions. Two pairs of primers specifically targeting methylated or unmethylated CpG island of host *SOCS-1* gene were designed according to previous studies (Methylation primer pair: M-*SOCS-1*-F: 5′-GAGTATTCGCGTGTATTTTTAGG-3′, M-*SOCS-1*-R: 5′-CGACACAACTCCTACAACGACCG-3′; Un-methylation primer pair: U-*SOCS-1*-F: 5′-TGAGTATTTGTG TGTATTTTTAGG-3′, U-*SOCS-1*-R: 5′-CAACACAACTCCT ACAACAACCA-3′) (Ko et al., 2008). Each sample was detected for methylated and unmethylated CpG island, respectively, with the same amount of template. The reaction was performed using Fast Start Universal SYBR Green Master in LightCycler 96 (Roche diagnostic, GmbH). It started with pre-incubation at 95°C for 600 s, followed by 35 cycles at 95°C for 10 s, 60°C for 10 s, and 72°C for 40 s. After melting and cooling, the Cq values of methylated and unmethylated CpG island were obtained to calculate the relative methylation level of the CpG island of the host *SOCS-1* gene.

### Demethylation of the Suppressor of Cytokine Signaling-1 Gene in the Host Gene

The DNA methylation inhibitor 5-aza-2-deoxycytidine (DAC) (Sigma, St. Louis, MO, United States), clinically called Decitabine, was applied to demethylate *SOCS-1* gene in host cells at a concentration of 1.0 μM and for 4 days of treatment according to the previous report (Galm et al., 2003). The expression of SOCS-1 in cells was detected through both Western blot and immunocytochemistry.

### Statistical Analysis

Each experiment was repeated three times. All the data from three independent experiments were included for the final statistical analysis. Data were reported as the mean ± standard deviation for normal and median (interquartile range) for non-normal continuous variables. Measurement data were first analyzed for normality before being analyzed with a Student’s *t*-test. Non-parametric alternatives (Mann–Whitney U) were used for non-normal distributions. A Wilcoxon paired test was performed to detect the relationship between viral load and SOCS-1 expression in tissues. Multiple linear regression was used to model the effect of possible factors on the expression of SOCS-1, and statistical power was calculated. All CIs and resulting *p*-values were two-sided. Values were supposed to be statistically significant at *p* < 0.05. The analysis was processed using SPSS 18.00.

### Ethics Statements

These tissue samples and serum samples were obtained for medical or forensic purpose other than our study originally. Samples of HCC tissues were obtained according to surgical resection of tumors. Normal tissues were originally prepared for medicolegal expertise. Serum samples were obtained from CHC patients. Also, they were extra samples originally used for diagnosis of HCV infection. Written informed consent was signed by patients or their legally designated relatives, and additional approvals were obtained for further use of the samples for investigation purpose. All the procedures were approved and supervised by the Ethics Committee of West China Hospital, Sichuan University.

## Results

### Expression of Suppressor of Cytokine Signaling-1 in HCV-HCC Tissues

We retrieved data of 11 patients with positive serum anti-HCV or HCV RNA and diagnosis of “HCC” recorded in the pathologic specimen bank. Among them, full sets of tissues (HCC, adjacent, and distal) were obtained from seven patients and HCC tissues only were obtained from four patients. However, negative HCV core protein was found in one HCC tissue through IHC. Therefore, the enrolled tissues included 10 HCV-HCC tissues, seven adjacent tissues, and seven distal tissues. Correspondingly, 16 normal liver tissue sections were obtained as control.

**FIGURE 1:**
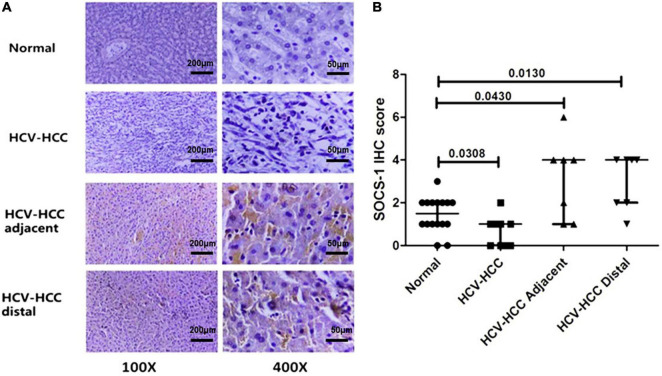
Immunohistochemical staining of suppressor of cytokine signaling 1 (SOCS-1) in different kinds of liver tissues. **(A)** Representative images of stained SOCS-1 in normal liver tissue, HCV–HCC tissues, HCV–HCC adjacent tissue, and HCV–HCC distal tissue (scale bar = 50 μm in 400× images, 200 μm in 100× images). **(B)** Axiotis scores of SOCS-1 expression in liver tissues. The enrolled tissues included 10 HCV-HCC tissues, seven adjacent tissues, and seven distal tissues. Also, 16 normal liver tissue sections were obtained as control (Mann–Whitney *U* test).

After SOCS-1 was stained (Figure 1A) and scored in tissues, it turned out that the median (interquartile range) expression score in HCV-HCC tissues was 0.5 (1) (Figure 1B). The median score in the normal tissue was 1.5 (1), whereas the median scores in the adjacent and distal tissues were 4 (3) and 4 (2), respectively (Figure 1B). The HCV-HCC tissues exhibited a lower expression of SOCS-1 than normal tissues (*p* = 0.0308). But the adjacent and distal tissues showed higher expression of SOCS-1 (*p* = 0.0430 and 0.0130, respectively; Figure 1B). The polarized alternation of SOCS-1 in HCV-HCC tissues and adjacent/distal tissues appeared to be different from our prior expectation about the impact of the virus on SOCS-1. However, we observed that the inflammatory infiltration was different between HCC tissues and the adjacent/distal tissues. As high histological inflammation can increase SOCS-1 expression by inducing infiltration of inflammatory cells, the interaction between HCV and SOCS-1 may be strongly influenced by histological inflammation.

### Correlation Between Viral Load and Suppressor of Cytokine Signaling-1 Expression in Tissues

**FIGURE 2:**
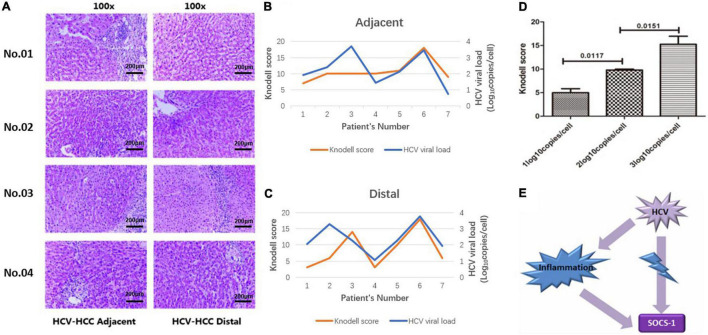
Histological inflammation in non-HCC liver tissues with HCV infection (including adjacent and distal tissues). **(A)** Representative image of HE-stained non-HCC liver tissue with HCV infection (scale bar = 200 μm). **(B)** The correlation between HCV viral load and Knodell scores in adjacent tissues of HCV-HCC. **(C)** The correlation between HCV viral load and Knodell scores in distal tissues of HCV-HCC. **(D)** Knodell scores in non-HCC tissues with different viral loads. Knodell score in tissues with a high viral load was higher compared to tissues with a low viral load. **(E)** Sketch map: Histological inflammation would induce expression of SOCS-1. HCV infection-induced inflammation may interfere with the correlation between HCV and SOCS-1 expression (Mann–Whitney *U* test).

Next, we analyzed the correlation between virus and SOCS-1 expression in tissues with the same histological inflammation to eliminate inflammatory effects on SOCS-1 expression. However, the Knodell score was not applicable in cancer tissues because their histological structure was not well preserved. This analysis could only be implemented in adjacent and distal tissues. After HE staining (Figure 2A), the seven adjacent tissues and seven distal tissues exhibited different Knodell scores ranging from 3 to 18. The Knodell score and HCV viral load in the seven adjacent tissues and seven distal tissues were quantified, and the distribution is shown in Figures 2B,C. The lowest Knodell scores in adjacent and distal tissues were 7 and 3, respectively, whereas the highest score was 18 in both groups (Figures 2B,C). From Figures 2B,C, it seems that Knodell score is positively correlated with HCV viral load in adjacent and distal tissues.

Then, samples were divided into three groups based on the viral load, and we observed that the median Knodell scores in group “HCV RNA < 2 log_10_ copies/cell,” “2 ≤ HCV RNA < 3 log_10_ copies/cell,” and “HCV RNA > 3 log_10_ copies/cell” were 6 (3), 10 (0.5), and 16 (6.25), respectively, with a significant difference. It was obvious that a higher viral load was correlated with a higher Knodell score (Figure 2D). As high histological inflammation can increase SOCS-1 expression, this interference factor, histological inflammation, should be considered in the analysis of the direct correlation between HCV and SOCS-1 (Figure 2E).

**FIGURE 3:**
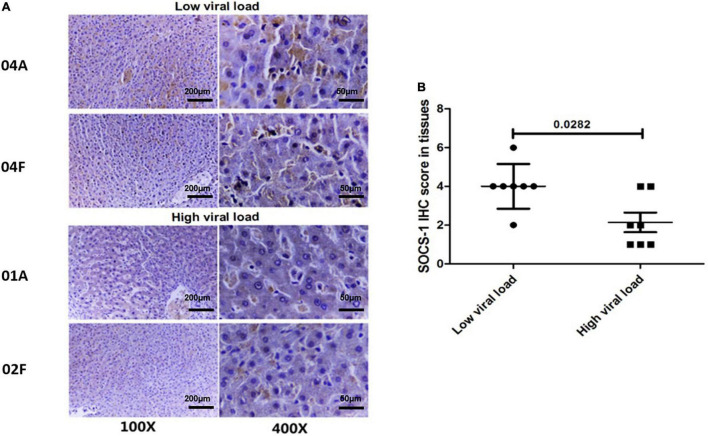
Tissues with a high viral load exhibit a lower level of SOCS-1 expression when they have similar histological inflammation. **(A)** Representative images of SOCS-1 expression in adjacent and distal tissues with different viral loads. “04, 01, and 02” represent patients’ numbers. “A” represents adjacent tissues. “F” represents distal tissues (scale bar = 50 μm in 400× images, 200 μm in 100× images). **(B)** Axiotis score of SOCS-1 expression in adjacent and distal tissues with different viral loads (Wilcoxon paired test).

Therefore, we paired the 14 adjacent and distal tissues according to their Knodell scores and divided those with similar Knodell scores into two groups based on the viral load. With the Wilcoxon paired test, we showed that the SOCS-1 expression in the high viral load group was much lower than the other groups (Figures 3A,B). The result suggested that HCV infection has a direct inhibitory effect on SOCS-1 expression.

In addition, we also analyzed the effects of several possible factors on the expression of SOCS-1 in non-HCC tissues, such as age, gender, histological inflammation, location (adjacent and distal tissues), and HCV viral load in tissues. Multiple linear regression analysis showed that the patients’ age had a significant impact on the expression of SOCS-1 in non-HCC liver tissues (*p* < 0.05; Table 1). The patients’ gender, histological inflammation, location, and HCV viral load had no significant impact on the expression of SOCS-1 (*p* > 0.05).

**TABLE 1:** Regression analysis of the effects on SOCS-1 expression in non-HCC tissue sections.

	Univariate			Multivariate		
	Coefficient	*P* value	95% CI	Coefficient	*P* value	95% CI
(Constant)	−0.343*	0.752*	−2.65 to 1.964*	–0.988	0.706	−6.812 to 4.835
Gender	–0.091	0.932	−2.369 to 2.187	0.235	0.815	−2.001 to 2.471
Age	0.061	0.006	0.022 to 0.100	0.064	0.035	0.006 to 0.123
Location	–0.143	0.870	−2.010 to 1.725	–0.143	0.865	−2.017 to 1.732
Knodell score	0.104	0.275	−0.094 to 0.301	–0.037	0.763	−0.310 to 0.236
HCV viral load	0.456	0.543	−1.132 to 2.043	0.307	0.735	−1.710 to 2.324

*This parameter was calculated when predictor variates were age and constant.

### Effect of HCV on Suppressor of Cytokine Signaling-1 Expression in HCV-Replicating Cell Lines

HCV replicon JFH-1 and HCV replicon cell Huh7.5.1 were generated to investigate the possible inhibitory effect from HCV to SOCS-1 expression. As a classic HCV replication system, HCV replicon was widely used in the study on HCV life cycle and pathogenesis. Moreover, the interference factor, infiltration of inflammatory cells, is absent in the cell culture system. These characteristics make it a better model to explore the effect of HCV on SOCS-1.

Each 100 μl cultural supernatant containing the cell cultural HCV particles (HCVcc) around 7 log_10_ copies/ml was added into the 2-ml culture medium in each well of the six-well plates. After 3 days, the viral load in the cultural supernatant was detected, and the viral load in host cells on day 3 was identified. The median viral load in host cells was 2.25 ± 0.39 log_10_ copies.

**FIGURE 4:**
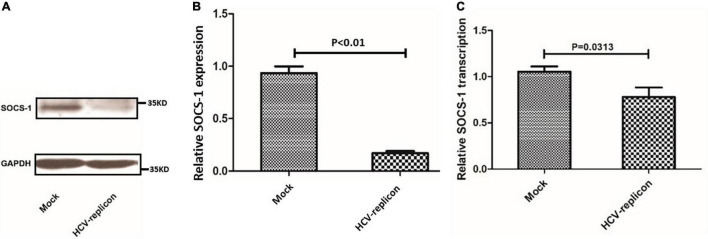
Suppressor of cytokine signaling 1 protein and mRNA expression in Huh7.5.1 cells treated with regular cultural supernatant from other Huh7.5.1 cells (Mock) and treated with cultural supernatant containing HCVcc generated from HCV replicon JFH-1 (HCV-replicon). **(A)** Western blot image of SOCS-1 in cells with and without HCVcc infection. **(B)** SOCS-1 protein level in cells with and without HCVcc infection. **(C)** *SOCS-1* mRNA level in cells with and without HCVcc infection (Student’s *t*-test).

Then, SOCS-1 expression in HCV-replicating cell lines was analyzed. Compared with the original cell line Huh7.5.1, the SOCS-1 expression was reduced by nearly 80% in HCV-replicating cell lines (Huh7.5.1 cells with HCVcc-JFH1) (Figures 4A,B). The decrease of SOCS-1 expression in HCV-replicating cell lines confirmed the negative regulatory effect from HCV to SOCS-1 *in vitro*. Furthermore, when we studied the transcription of *SOCS-1*, we found that in HCV-replicating cell lines, it was downregulated as well (Figure 4C).

### HCV-Regulating Suppressor of Cytokine Signaling-1 Expression in HCV-Replicating Cell Lines

**FIGURE 5:**
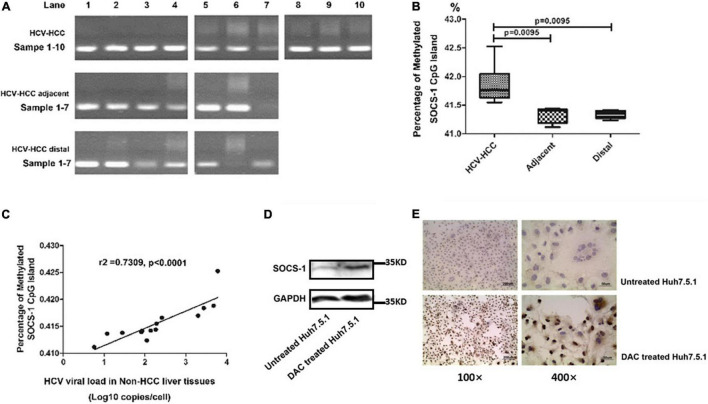
The methylation of the CpG island in the *SOCS-1* gene promoter affects SOCS-1 expression in tissues and cell lines. **(A)** The methylation of the CpG island in *SOCS-1* gene promoter in HCV-HCC tissues and related tissues. **(B)** The statistical diagram of the methylation of the CpG island in *SOCS-1* gene promoter in three kinds of tissues. **(C)** The correlation between *SOCS-1* gene methylation and HCV viral load in non-HCC liver tissues. **(D)** The Western blot of SOCS-1 expression in HCVcc-infected Huh7.5.1 cells treated by DAC. **(E)** The ICC image of SOCS-1 expression in HCVcc-infected Huh7.5.1 cells treated by DAC (Mann–Whitney *U* test).

The hypermethylation of the CpG island of host *SOCS-1* gene was one of the reported mechanisms to downregulate SOCS-1 expression. As SOCS-1 is decreased at both mRNA and protein levels, we intended to understand the effect of DNA methylation on *SOCS-1* downregulation. We applied MSP to detect and quantify the methylation status of CpG island of host gene, and a positive methylated SCOS-1 CpG island was identified in all 10 HCV-HCC tissues and most of the non-HCC tissues with HCV infection. In two non-HCC tissues, there seemed to be no band of *SOCS-1* CpG methylation, but we still detected slightly positive *SOCS-1* CpG methylation through real-time PCR (Figure 5A).

The unmethylated CpG island of *SOCS-1* was also quantified to calculate the percentage of methylated *SOCS-1* CpG island through a formula “Relative quantification of methylated CpG island/(relative quantification of methylated CpG island + relative quantification of unmethylated CpG island).” When the methylation level of host genes in cancer tissues was analyzed, it is slightly but significantly higher than that in the non-cancer tissues (adjacent and distal tissues) (42.0 vs. 41.4% and 41.3%) (Figure 5B). Although a lot of other interference factors such as histological inflammation, fibrocytes, adipocytes, vascular endothelial cells, and other cells were involved, the differences seemed to be limited. It is still suggested that the lower expression of SOCS-1 in HCV-HCC tissues may result in part due to the hypermethylation of the CpG island in *SOCS-1* gene.

To understand whether HCV influenced the CpG methylation, we analyzed the correlation between the methylation level of *SOCS-1* and HCV viral load in the non-caner tissues. Interestingly, a moderately positive correlation was found in these tissues (*r*^2^ = 0.7309, *p* < 0.0001; Figure 5C). Although along with interference factors such as inflammatory cell infiltration and fibrocytes, pure genomic DNA of hepatocytes could not be detected. This result still strongly implied an underlying effect of HCV on gene methylation.

Moreover, when we treated the HCV infected cells with 5-aza-2-deoxycytidine (DAC), a DNA methylation inhibitor, the restored expression of SOCS-1 was successfully detected through both Western blot and ICC (Figures 5D,E).

## Discussion

The development of HCC requires activation of oncogenes, suppression of antitumor factors, and alternation of the cellular microenvironment (Hanahan and Weinberg, 2011). Subsequently, cellular genomic DNA becomes unstable and the cell becomes immortal. HCV exactly regulated various oncogenes and antitumor factors as a tumor-promoting virus (Hibner and Gregoire, 2015). SOCS-1 was one of these antitumor factors. Tadamitsu et al. knocked it out of HCC cells and showed that deficiency of SOCS-1 resulted in further malignant transformation of these cells, including paramorphia and overproliferation (Kishimoto and Kikutani, 2001).

pDC-mediated antigen presentation activates endogenous IFN synthesis and the JAK/STAT pathway and accelerates expression of antiviral proteins. As a member of ISGs, SOCS-1 expression was increased along with other antiviral proteins. It acted as a negative regulatory factor to suppress the activation of JAK/STAT pathways (Levy and Darnell, 2002; Steen and Gamero, 2013). To some degree, this mechanism decreases tumor-promoting inflammation and helps the maintenance of the internal environment. However, JAK/STAT was not the only pathway through which the virus controlled SOCS-1 expression. In a study concerning respiratory syncytial virus (RSV), the virus could directly induce overexpression of SOCS-1 to weaken the innate immunity (Xu et al., 2014). HCV may also regulate SOCS-1 expression through other ways. Miyoshi et al. established expression of HCV core protein in transgenic mice and HepG2 cells. They found an inhibitory effect from HCV core protein on SOCS-1 expression (Miyoshi et al., 2005). The only drawback was that the core protein-expressing models lacked the completed structure of viral particles. Also, it could not fully clarify the impact of viruses on host cytokines in these models. So, we applied HCV-infected liver tissues for the investigation, and we used a more classic and more convincing model, the replicon model, to learn the actual effect of the virus on SOCS-1. In tissues, we applied the HAI score and Wilcoxon paired test to balance the influence of histological inflammation and found a negative correlation between viral load and SOCS-1 expression. Besides, multiple linear regression analysis showed that the patients’ age had a significant impact on the expression of SOCS-1 in non-HCC liver tissues. The patients’ gender, histological inflammation, location, and HCV viral load had no significant impact on the expression of SOCS-1. It is consistent with the fact that there is interaction between the virus, inflammation, tumorigenesis, and SOCS-1 expression. Only age is the independent impact factor on SOCS-1 expression, leading us to further exploration on aging-related signaling. In HCV replicon models, an environment without exogenous interferon or endogenous interferon from immune cells, we validated the negative regulatory effect of HCV on SOCS-1. Our findings provide consistent and strong evidence that HCV can downregulate the expression of SOCS-1 directly. Whether it is inhibited by HCV core protein as reported by Miyoshi still needs further confirmation by subsequent studies.

The level of cytokines was regulated at different levels. Epigenetic modification at the gene level, transcriptional activation and inhibition at the mRNA level, posttranscriptional stage, and translation and degradation at protein level are all common regulable ways. As for SOCS-1, the cause of its low expression lies in the abnormal methylation of CpG island next to the promoter of its coding gene, namely, DNA methylation-induced gene silencing. Yoshikawa et al. reported such abnormal methylation in HCC cell lines. They also conducted a small analysis with limited clinical samples to confirm the finding (Yoshikawa et al., 2001). The result showed that 65% of the enrolled liver tissues from HCC patients with various causes had aberrant promoter methylation. However, it could only suggest a correlation between HCC and aberrant promoter methylation but insufficient to connect HCV infection to it. Similarly, Eric et al. conducted another study to detect the promoter methylation status of tumor-related cytokines in HCV-HCC tissues. They enrolled 43 cancer tissues and 45 non-cancer tissues, and 10 normal liver tissues as control. In the end, aberrant promoter methylation was found in 91% of the tissues, regardless of cancer or non-cancer tissues. Interestingly, no promoter methylation of *SOCS-1* was found in normal liver tissues. It suggested that HCV infection was a critical factor in inducing methylation of *SOCS-1* (Formeister et al., 2010). This study provides hints to connect HCV infection to SOCS-1, but more valid evidence should be provided. Therefore, we did further investigation in our study. We collected identified HCV-infected tissues and excluded cancer tissue to implement the analysis. A positive correlation between HCV viral load and the methylation degree of promoters was identified. Together with the restored SOCS-1 expression from DAC treatment in HCV-infected cells, our finding successfully proved that HCV infection is correlated with DNA hypermethylation, which is involved in the downregulation of SOCS-1 expression.

There are some potential limitations to this study. First, the level of SOCS-1 in liver tissues was detected on protein expression by IHC. Even though the mRNA level in cell models was determined to partly support the findings on gene methylation and reveal the possible change in mRNA level in tissues, further study on post-transcriptionally regulatory or translational/posttranslational signaling pathways is still needed. Besides, results from MSP analyzes revealed that the lower expression of SOCS-1 in HCV-HCC tissues may result from the hypermethylation of CpG islands in the *SOCS-1* gene. Although the differences among the three groups are slight but significant (Figure 5B), the sample sizes are still need to be expanded. Previous studies have also indicated that CpG island methylation in various carcinomas such as hepatocellular carcinoma, multiple myeloma, acute myeloid lymphoma, and colorectal cancer is responsible for silencing the *SOCS-1* gene (Chen et al., 2003; Galm et al., 2003; Nagai et al., 2003; Tischoff et al., 2007; Kang et al., 2016). As we found the different alternations on mRNA and protein level, there might be a new field of posttranscriptional or translational/posttranslational regulation to be explored. Many researches have demonstrated that downregulation of SOCS-1 could also come from posttranscriptional stages. For example, Song et al. (2021) demonstrated that the core miRNA biogenesis and targeting machinery were essential for the IFNγ-activated JAK-STAT signaling and antigen presentation in cancer cells, largely by controlling miR-155-targeted silencing of *SOCS-1*. In smoke inhalation-induced lung injury, miRNA-155 was involved in the inflammatory response by inhibiting the expression of SOCS-1 (Zhang et al., 2020). Wang et al. (2018) also illustrated that HBeAg augmented the expression of miR-155 to promote inflammatory cytokine production by inhibiting the expression of SOCS-1. Besides, it is reported that miR-19s and miR-30a can also negatively regulate SOCS-1 (Marioni et al., 2016; Yuan et al., 2019). It illustrates that post-transcription is also an important mechanism in the downregulation of SOCS-1. In addition, Gregorieff et al. (2000) reported that the expression of SOCS-1 can be repressed at the level of translation initiation mediated by the 5′ untranslated region of *SOCS-1*. However, the translational or posttranslational regulation of SOCS-1 in cancers has rarely been reported. Therefore, more studies are needed to detect whether epigenetic regulation is the most important regulatory pathway of HCV on SOCS-1 and uncover the deeper mechanisms.

## Conclusion

In spite of inflammatory cell infiltration, HCV-HCC tissues still had low expression of SOCS-1. The downregulation may result from HCV infection. Higher HCV viral load may lead to lower expression of SOCS-1 in hepatocytes. The follow-up study proved that the downregulatory effect was at an epigenetic level, in which HCV infection could induce gene methylation in the CpG island of *SOCS-1* gene promoters to suppress its expression. The relationship between HCV and *SOCS-1* gene methylation may be a possible factor contributing to HCC genesis after HCV elimination and is worth for further investigation.

## Data Availability Statement

The original contributions presented in this study are included in the article/Supplementary Material, further inquiries can be directed to the corresponding author.

## Ethics Statement

The studies involving human participants were reviewed and approved by Ethics Committee of West China Hospital, Sichuan University. The patients/participants provided their written informed consent to participate in this study.

## Author Contributions

HT proposed the conception and designed the study, provided fund support, and approved the submitted version. ML, LD, XC, MY, JS, and YS implemented the study and collected the data. ML and LD analyzed the data and drafted the manuscript. HY and HT revised the manuscript. All authors contributed to the article and approved the submitted version.

## Conflict of Interest

The authors declare that the research was conducted in the absence of any commercial or financial relationships that could be construed as a potential conflict of interest.

## Publisher’s Note

All claims expressed in this article are solely those of the authors and do not necessarily represent those of their affiliated organizations, or those of the publisher, the editors and the reviewers. Any product that may be evaluated in this article, or claim that may be made by its manufacturer, is not guaranteed or endorsed by the publisher.

## References

[B1] Casanova Y. S. Boeira Tda R. Sisti E. Celmer A. Fonseca A. S. Ikuta N. (2014). A complete molecular biology assay for hepatitis C virus detection, quantification and genotyping. *Rev. Soc. Bras. Med. Trop.* 47 287–294. 10.1590/0037-8682-0040-2014 25075478

[B2] Chen C. Y. Tsay W. Tang J. L. Shen H. L. Lin S. W. Huang S. Y. (2003). SOCS1 methylation in patients with newly diagnosed acute myeloid leukemia. *Genes Chromosomes Cancer* 37 300–305. 10.1002/gcc.10222 12759928

[B3] Chen S. L. Morgan T. R. (2006). The natural history of hepatitis C virus (HCV) infection. *Int. J. Med. Sci.* 3 47–52. 16614742 10.7150/ijms.3.47 PMC1415841

[B4] Corey K. E. Mendez-Navarro J. Gorospe E. C. Zheng H. Chung R. T. (2010). Early treatment improves outcomes in acute hepatitis C virus infection: a meta-analysis. *J. Viral Hepat.* 17 201–207. 10.1111/j.1365-2893.2009.01167.x 19674285 PMC3769693

[B5] Date T. Miyamoto M. Kato T. Morikawa K. Murayama A. Akazawa D. (2007). An infectious and selectable full-length replicon system with hepatitis C virus JFH-1 strain. *Hepatol. Res.* 37 433–443. 10.1111/j.1872-034X.2007.00056.x 17437527

[B6] Du L. Y. Cui Y. L. Chen E. Q. Cheng X. Liu L. Tang H. (2014). Correlation between the suppressor of cytokine signaling-1 and 3 and hepatitis B virus: possible roles in the resistance to interferon treatment. *Virol. J.* 11:51. 10.1186/1743-422X-11-51 24636575 PMC3995528

[B7] Du L. Y. Tang H. (2016). miRNA antagonism and direct-acting antivirals: Could this be a novel combination treatment against HCV? *Future Virol.* 11 753–756.

[B8] Formeister E. J. Tsuchiya M. Fujii H. Shpyleva S. Pogribny I. P. Rusyn I. (2010). Comparative analysis of promoter methylation and gene expression endpoints between tumorous and non-tumorous tissues from HCV-positive patients with hepatocellular carcinoma. *Mutat. Res.* 692 26–33. 10.1016/j.mrfmmm.2010.07.013 20736025 PMC2948626

[B9] Galm O. Yoshikawa H. Esteller M. Osieka R. Herman J. G. (2003). SOCS-1, a negative regulator of cytokine signaling, is frequently silenced by methylation in multiple myeloma. *Blood* 101 2784–2788. 10.1182/blood-2002-06-1735 12456503

[B10] Goodman Z. D. (2007). Grading and staging systems for inflammation and fibrosis in chronic liver diseases. *J. Hepatol.* 47 598–607. 10.1016/j.jhep.2007.07.006 17692984

[B11] Gregorieff A. Pyronnet S. Sonenberg N. Veillette A. (2000). Regulation of SOCS-1 expression by translational repression. *J. Biol. Chem.* 275 21596–21604. 10.1074/jbc.M910087199 10764816

[B12] Hanahan D. Weinberg R. A. (2011). Hallmarks of cancer: the next generation. *Cell* 144 646–674. 21376230 10.1016/j.cell.2011.02.013

[B13] Hibner U. Gregoire D. (2015). Viruses in cancer cell plasticity: the role of hepatitis C virus in hepatocellular carcinoma. *Contemp. Oncol.* 19 A62–A67. 10.5114/wo.2014.47132 25691824 PMC4322526

[B14] Kang X. C. Chen M. L. Yang F. Gao B. Q. Yang Q. H. Zheng W. W. (2016). Promoter methylation and expression of SOCS-1 affect clinical outcome and epithelial-mesenchymal transition in colorectal cancer. *Biomed. Pharmacother.* 80 23–29. 10.1016/j.biopha.2016.02.011 27133036

[B15] Kishimoto T. Kikutani H. (2001). Knocking the SOCS off a tumor suppressor. *Nat. Genet.* 28 4–5. 10.1038/88244 11326261

[B16] Ko E. Kim S. J. Joh J. W. Park C. K. Park J. Kim D. H. (2008). CpG island hypermethylation of SOCS-1 gene is inversely associated with HBV infection in hepatocellular carcinoma. *Cancer Lett.* 271 240–250. 10.1016/j.canlet.2008.06.009 18639978

[B17] Levy D. E. Darnell J. E. Jr. (2002). Stats: transcriptional control and biological impact. *Nat. Rev. Mol. Cell Biol.* 3 651–662. 10.1038/nrm909 12209125

[B18] Liang L. B. Zhu X. Yan L. B. Du L. Y. Liu C. Liao J. (2016). Quantitative intrahepatic HBV cccDNA correlates with histological liver inflammation in chronic hepatitis B virus infection. *Int. J. Infect. Dis.* 52 77–82. 10.1016/j.ijid.2016.09.022 27686728

[B19] Marioni G. Agostini M. Cappellesso R. Bedin C. Ottaviano G. Marchese-Ragona R. (2016). miR-19a and SOCS-1 expression in the differential diagnosis of laryngeal (glottic) verrucous squamous cell carcinoma. *J. Clin. Pathol.* 69 415–421. 10.1136/jclinpath-2015-203308 26502748

[B20] Medvedev R. Ploen D. Hildt E. (2016). HCV and Oxidative Stress: implications for HCV Life Cycle and HCV-Associated Pathogenesis. *Oxid. Med. Cell. Longev.* 2016:9012580. 10.1155/2016/9012580 26955431 PMC4756209

[B21] Miyoshi H. Fujie H. Shintani Y. Tsutsumi T. Shinzawa S. Makuuchi M. (2005). Hepatitis C virus core protein exerts an inhibitory effect on suppressor of cytokine signaling (SOCS)-1 gene expression. *J. Hepatol.* 43 757–763. 10.1016/j.jhep.2005.03.028 16083990

[B22] Nagai H. Naka T. Terada Y. Komazaki T. Yabe A. Jin E. (2003). Hypermethylation associated with inactivation of the SOCS-1 gene, a JAK/STAT inhibitor, in human hepatoblastomas. *J. Hum. Genet.* 48 65–69. 10.1007/s100380300008 12601549

[B23] O’sullivan L. A. Liongue C. Lewis R. S. Stephenson S. E. M. Ward A. C. (2007). Cytokine receptor signaling through the Jak-Stat-Socs pathway in disease. *Mol. Immunol.* 44 2497–2506. 10.1016/j.molimm.2006.11.025 17208301

[B24] Qu Y. Dang S. Hou P. (2013). Gene methylation in gastric cancer. *Clin. Chim. Acta* 424 53–65. 23669186 10.1016/j.cca.2013.05.002

[B25] Rao H. Wei L. Lopez-Talavera J. C. Shang J. Chen H. Li J. (2014). Distribution and clinical correlates of viral and host genotypes in Chinese patients with chronic hepatitis C virus infection. *J. Gastroenterol. Hepatol.* 29 545–553. 10.1111/jgh.12398 24090188 PMC4272577

[B26] Rottapel R. Ilangumaran S. Neale C. La Rose J. Ho J. M. Nguyen M. H. (2002). The tumor suppressor activity of SOCS-1. *Oncogene* 21 4351–4362. 10.1038/sj.onc.1205537 12080466

[B27] Song T. Y. Long M. Zhao H. X. Zou M. W. Fan H. J. Liu Y. (2021). Tumor evolution selectively inactivates the core microRNA machinery for immune evasion. *Nat. Commun.* 12:7003. 10.1038/s41467-021-27331-3 34853298 PMC8636623

[B28] Steen H. C. Gamero A. M. (2013). STAT2 phosphorylation and signaling. *JAKSTAT* 2:e25790. 10.4161/jkst.25790 24416652 PMC3876438

[B29] Stone A. E. Giugliano S. Schnell G. Cheng L. Leahy K. F. Golden-Mason L. (2013). Hepatitis C virus pathogen associated molecular pattern (PAMP) triggers production of lambda-interferons by human plasmacytoid dendritic cells. *PLoS Pathog.* 9:e1003316. 10.1371/journal.ppat.1003316 23637605 PMC3630164

[B30] Tischoff I. HEngge U. R. Vieth M. Ell C. Stolte M. Weber A. (2007). Methylation of SOCS-3 and SOCS-1 in the carcinogenesis of Barrett’s adenocarcinoma. *Gut* 56 1047–1053. 10.1136/gut.2006.111633 17376806 PMC1955493

[B31] Wang W. W. Bian H. J. Li F. F. Li X. Zhang D. Sun S. H. (2018). HBeAg induces the expression of macrophage miR-155 to accelerate liver injury via promoting production of inflammatory cytokines. *Cell. Mol. Life Sci.* 75 2627–2641. 10.1007/s00018-018-2753-8 29349567 PMC11105519

[B32] Wang Z. Niu X. Q. Zhou W. W. Lu Q. Y. (2015). [Effects of DNMT1 Gene Silencing on Methylation of SOCS-1 Gene in Myeloma Cells]. *Zhongguo Shi Yan Xue Ye Xue Za Zhi* 23 713–717. 10.7534/j.issn.1009-2137.2015.03.022 26117023

[B33] Waris G. Ahsan H. (2006). Reactive oxygen species: role in the development of cancer and various chronic conditions. *J. Carcinog.* 5:14. 10.1186/1477-3163-5-14 16689993 PMC1479806

[B34] Xu X. Zheng J. Zheng K. Hou Y. Zhao F. Zhao D. (2014). Respiratory syncytial virus NS1 protein degrades STAT2 by inducing SOCS1 expression. *Intervirology* 57 65–73. 10.1159/000357327 24480984

[B35] Yoshikawa H. Matsubara K. Qian G. S. Jackson P. Groopman J. D. Manning J. E. (2001). SOCS-1, a negative regulator of the JAK/STAT pathway, is silenced by methylation in human hepatocellular carcinoma and shows growth-suppression activity. *Nat. Genet.* 28 29–35. 10.1038/ng0501-29 11326271

[B36] Yuan F. H. Chen Y. L. Zhao Y. Liu Z. M. Nan C. C. Zheng B. L. (2019). microRNA-30a inhibits the liver cell proliferation and promotes cell apoptosis through the JAK/STAT signaling pathway by targeting SOCS-1 in rats with sepsis. *J. Cell. Physiol.* 234 17839–17853. 10.1002/jcp.28410 30972748

[B37] Zhang Y. Xie Y. Zhang L. Zhao H. (2020). MicroRNA-155 Participates in Smoke-Inhalation-Induced Acute Lung Injury through Inhibition of SOCS-1. *Molecules* 25:1022. 10.3390/molecules25051022 PMC7179228 32106541

[B38] Zhong J. Gastaminza P. Cheng G. F. Kapadia S. Kato T. Burton D. R. (2005). Robust hepatitis C virus infection *in vitro*. *Proc. Natl. Acad. Sci. U.S.A.* 102 9294–9299. 10.1073/pnas.0503596102 15939869 PMC1166622

