# Supplementary material for: CpG Island Methylation of Suppressor of Cytokine Signaling-1 Gene Induced by HCV Is Associated With HCV-Related Hepatocellular Carcinoma
Source: Front Microbiol. 2022 Jun 6;13:679593. doi: 10.3389/fmicb.2022.679593 (PMC9207397; doi:10.3389/fmicb.2022.679593)

In the supplementary, 'A' is represented for adjacent tissues. 'F' is represented for distal tissues, and 'C' for cancer tissues.

Table-1

Old Number	New Number	Gender	Age	Stage	Symptom	Treatment	HCV RNA(IU/ml)	Core Antigen Level
6123C/A/F	01C/A/F	F	44	II～III	epigastric pain	NA	NA	NA
7118C/A/F	02C/A/F	M	20	II	abdominal pain	NA	NA	NA
7158C/A/F	03C/A/F	M	69	II	epigastric pain and jaundice	NA	1.23E+06	NA
10141C/A/F	04C/A/F	M	57	II～III	epigastric pain	NA	NA	NA
10217C/A/F	05C/A/F	M	69	II～III	epigastric pain	NA	NA	NA
11220C/A/F	06C/A/F	M	64	II	abdominal pain	NA	NA	NA
12793C/A/F	07C/A/F	F	71	II～III	epigastric pain	NA	NA	NA
4925C	08C	M	65	II	abdominal pain	NA	NA	NA
7180C	09C	M	67	II	epigastric pain	NA	NA	NA
9238C	10C	M	38	II	epigastric pain and jaundice	NA	NA	NA

Gender: "F" is represented for Female. "M" is represented for Male.

Table-2

Number	Viral Load (Log10 copies/cell)
01A	1.92
01F	2.04
02A	2.41
02F	3.3
03A	3.67
03F	2.26
04A	1.45
04F	1.08
05A	2.14
05F	2.28
06A	3.44
06F	3.78
07A	0.76
07F	1.93

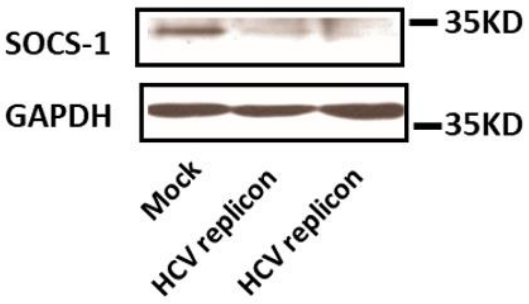
SOCS-1
GAPDH
35KD
35KD
Mock
HCV replicon
HCV replicon

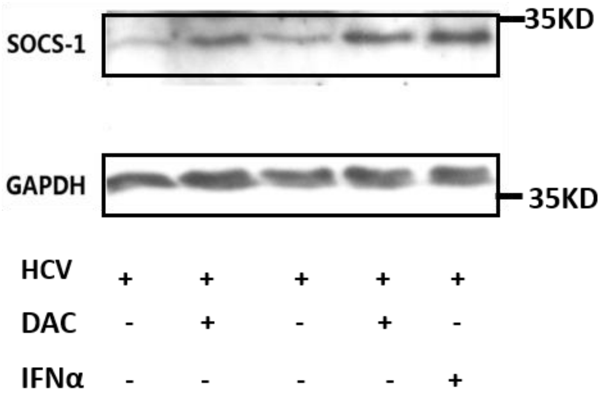
SOCS-1
GAPDH
35KD
35KD
HCV + + + + +
DAC - + - + -
IFNα - - - - +

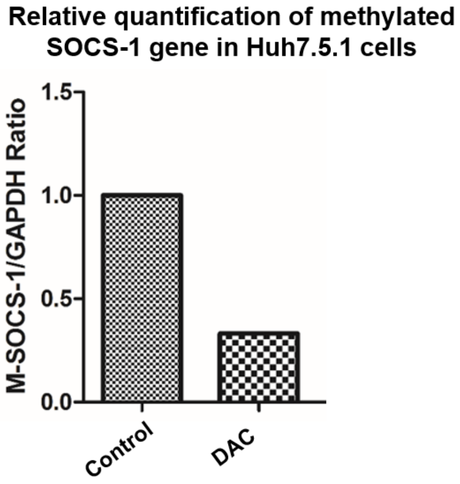
Relative quantification of methylated SOCS-1 gene in Huh7.5.1 cells
M-SOCS-1/GAPDH Ratio
1.5
1.0
0.5
0.0
Control
DAC

Immunohistochemical staining of SOCS-1 in different kind of liver tissues

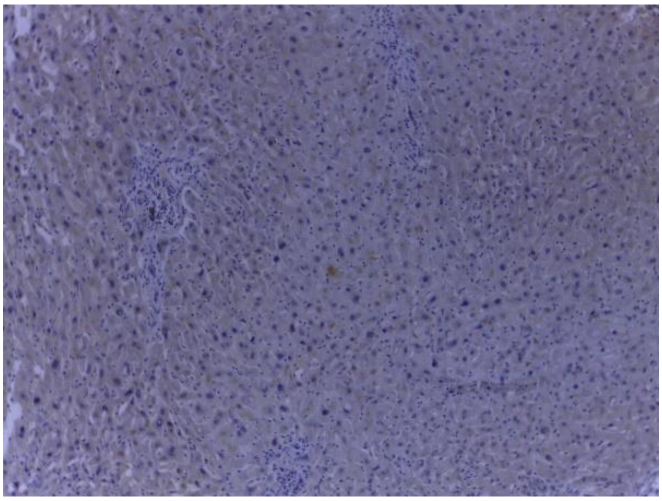

Normal-1

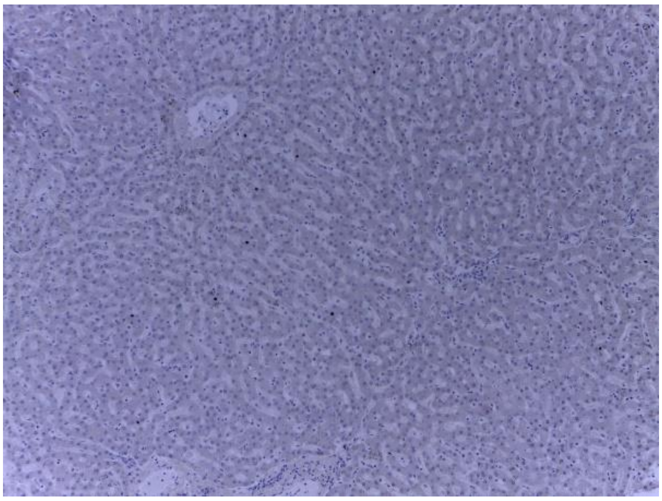

Normal-2

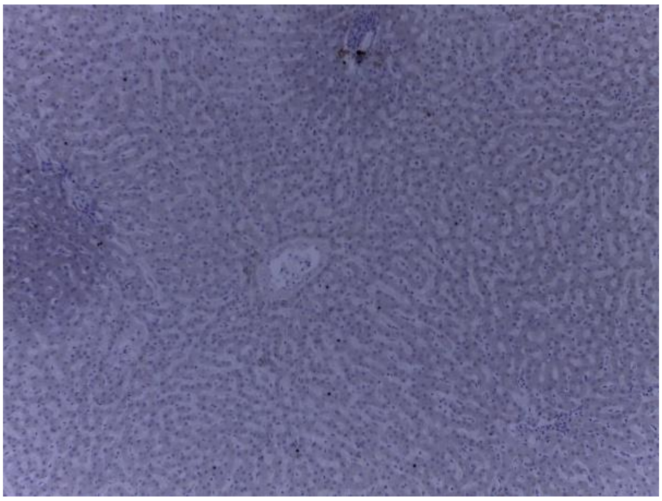

Normal-3

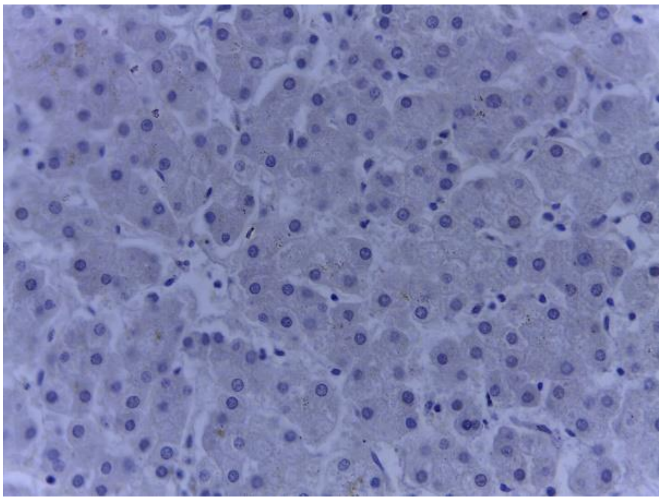

Normal-4

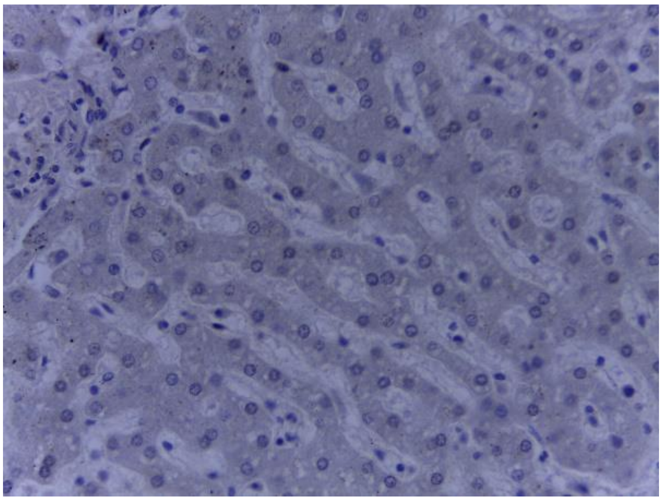

Normal-5

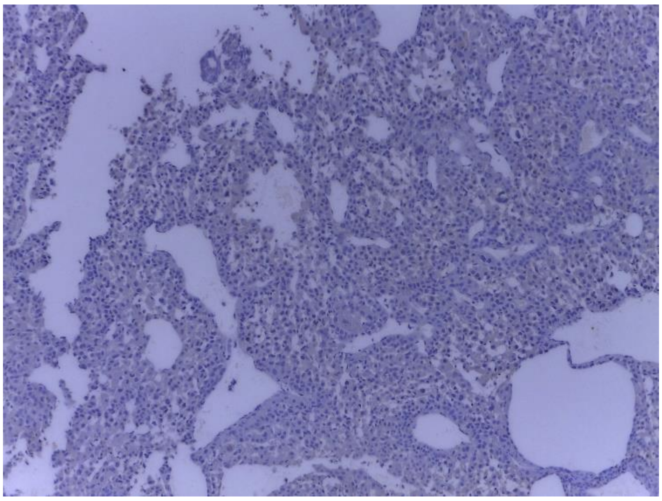

08C

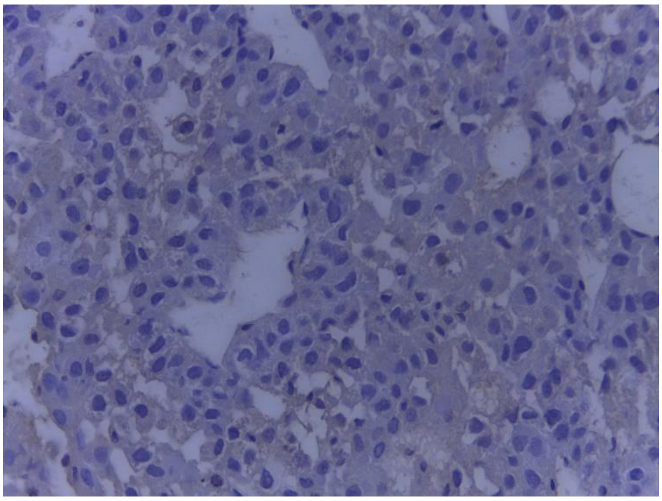

08C-1

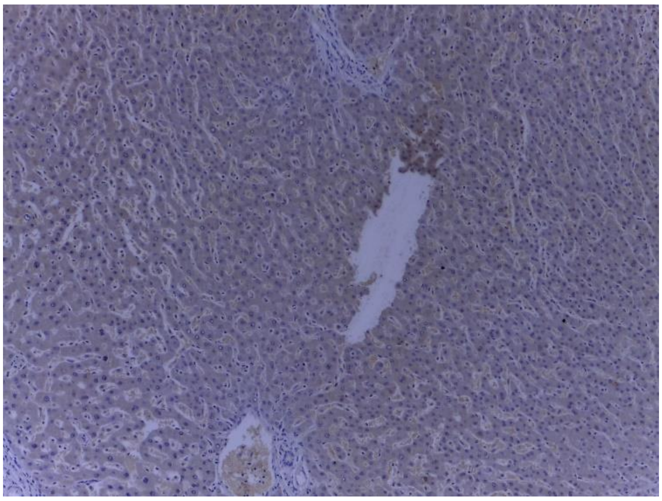

01A

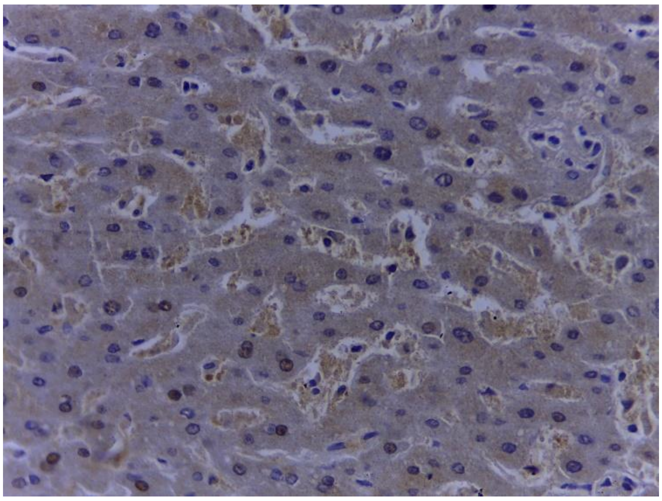

01A-1

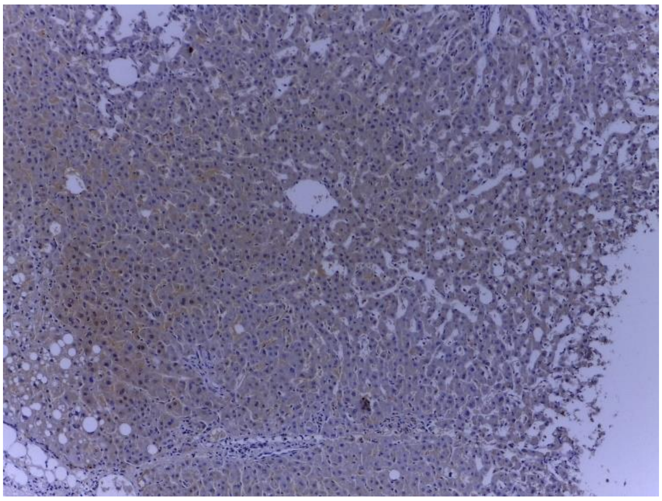

01F

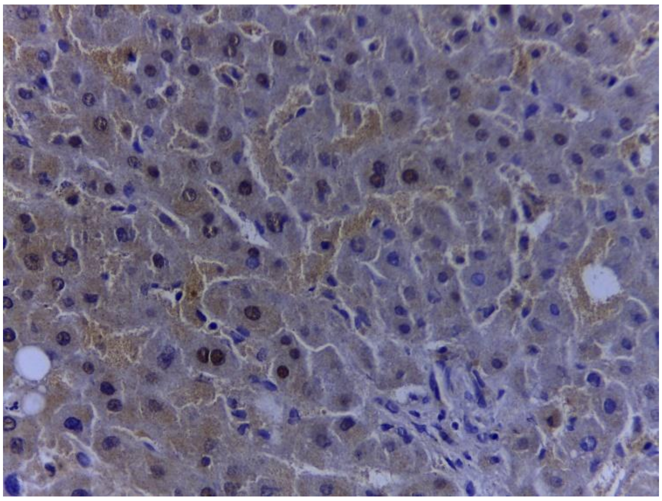

01F-1

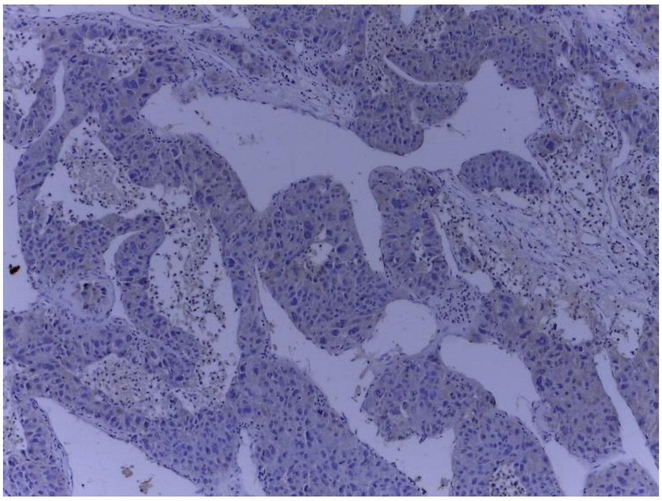

01C

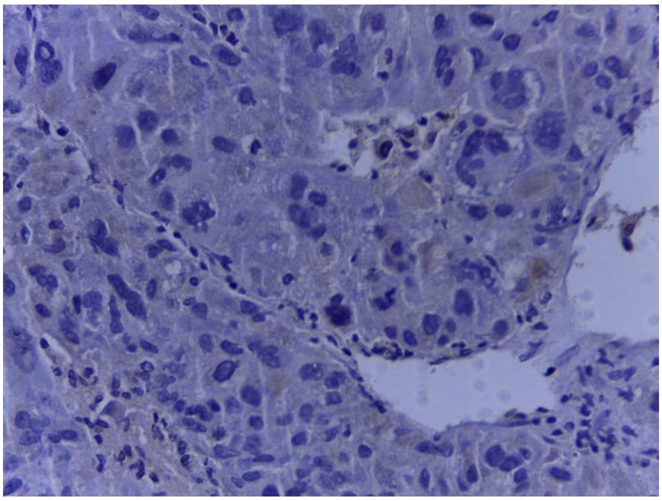
01C-1

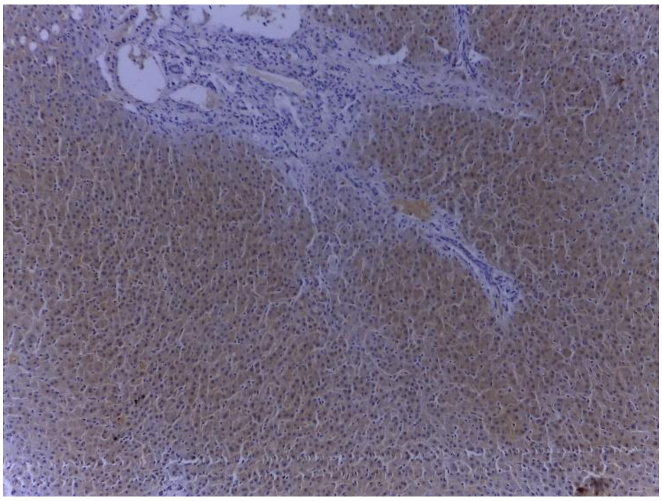
02A

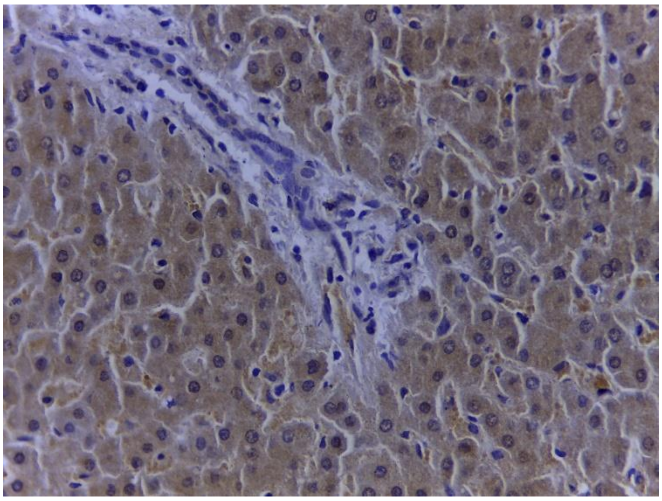
02A-1

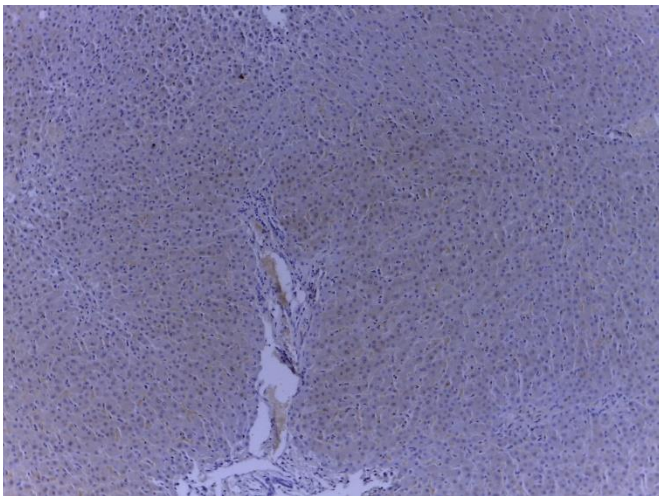
02F

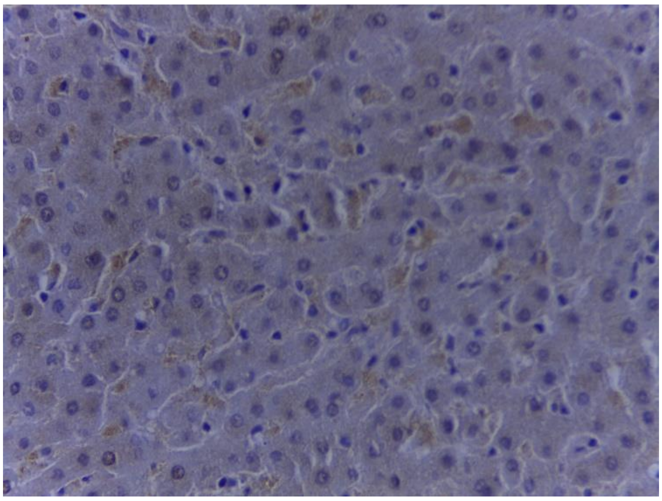
02F-1

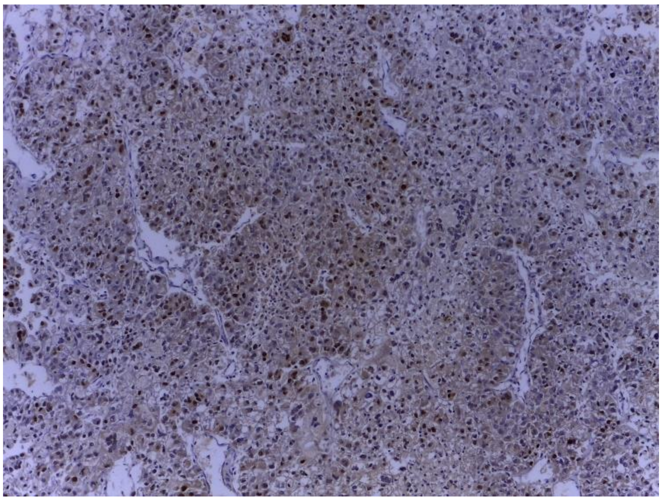
02C

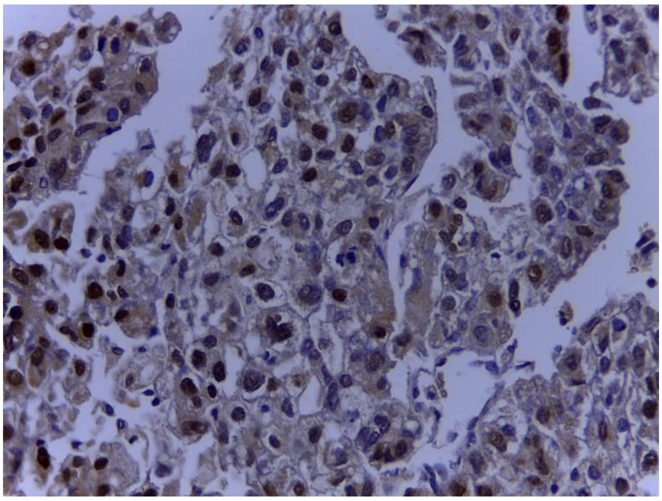

02C-1

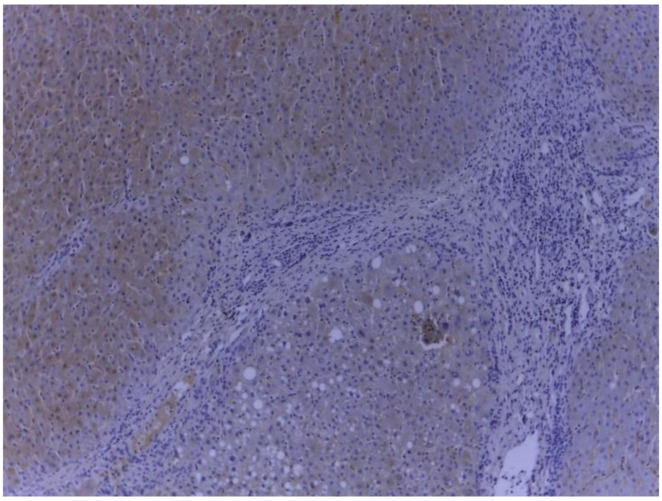

03A

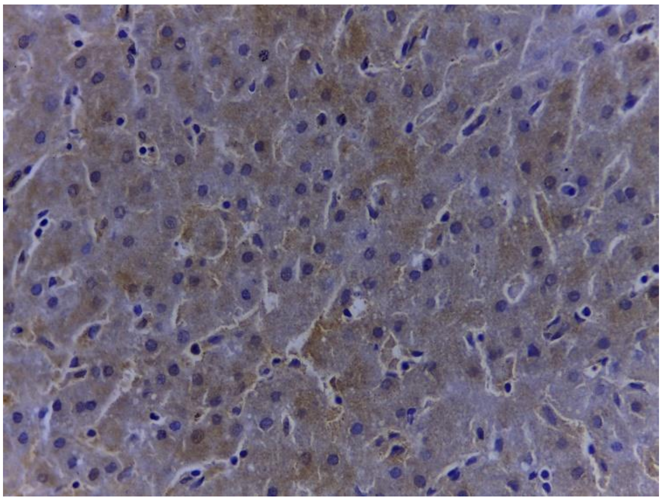

03A-1

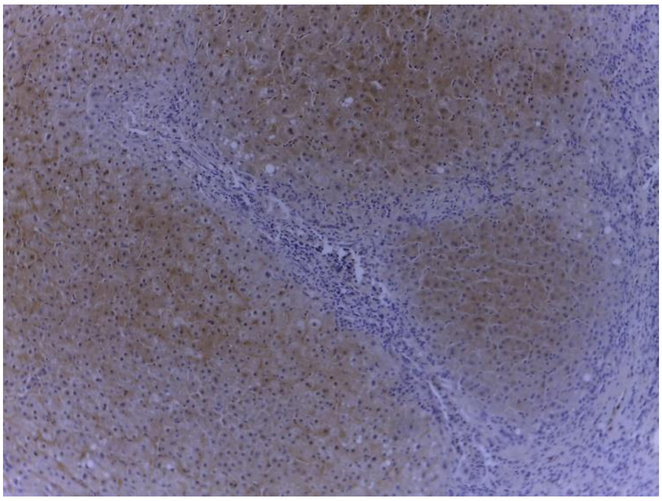

03F

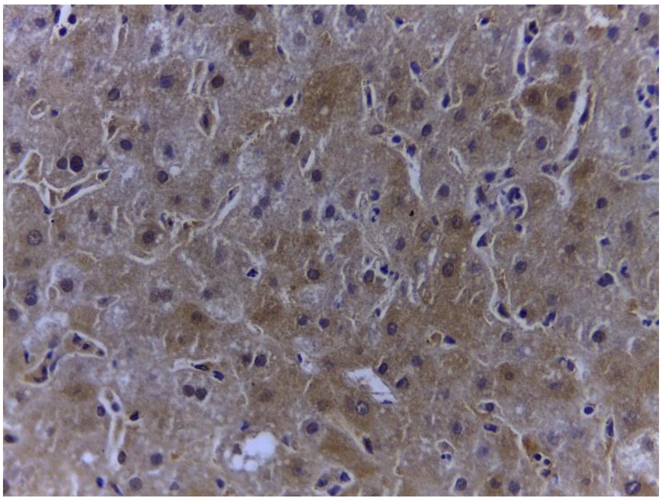

03F-1

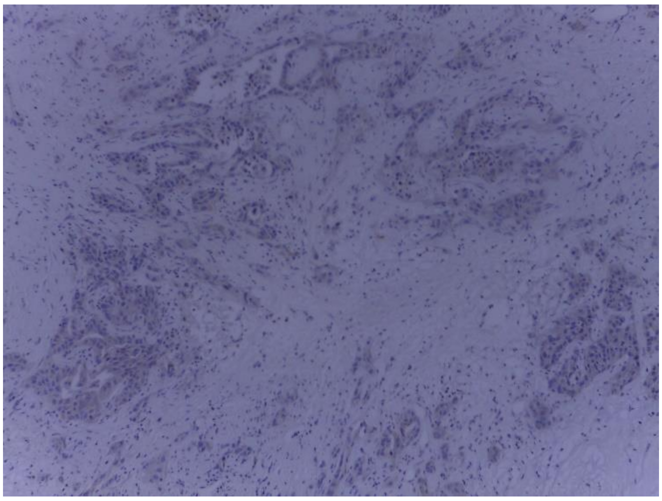

03C

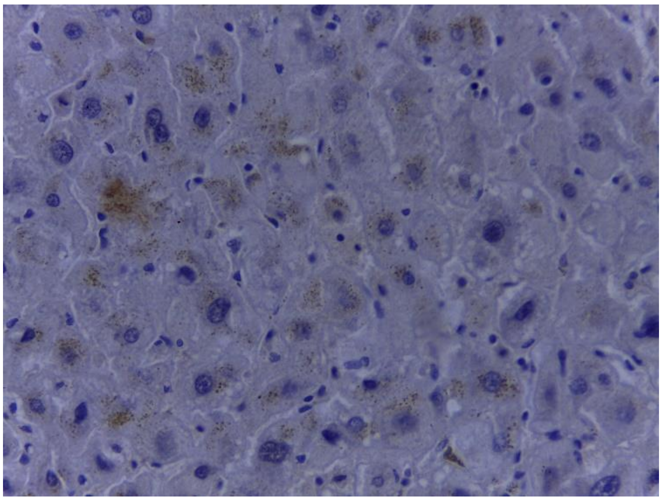

03C-1

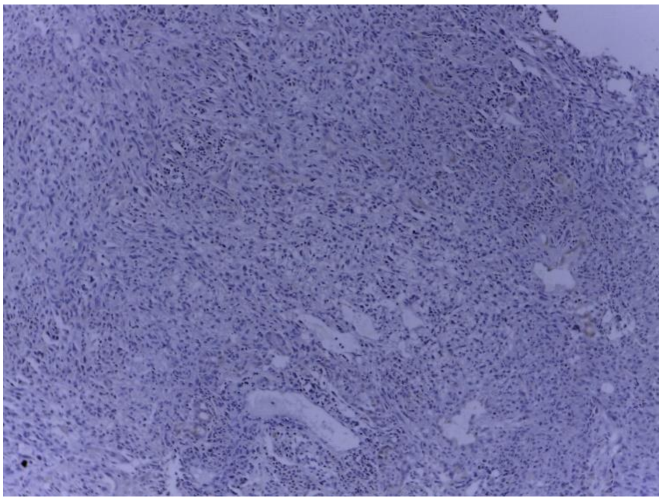

09C

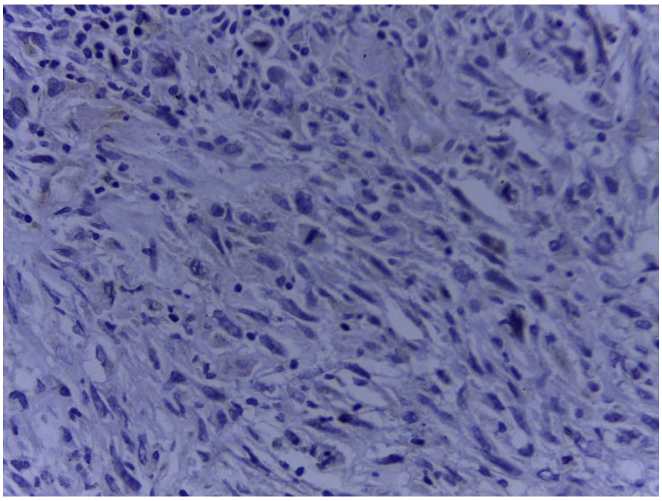

09C-1

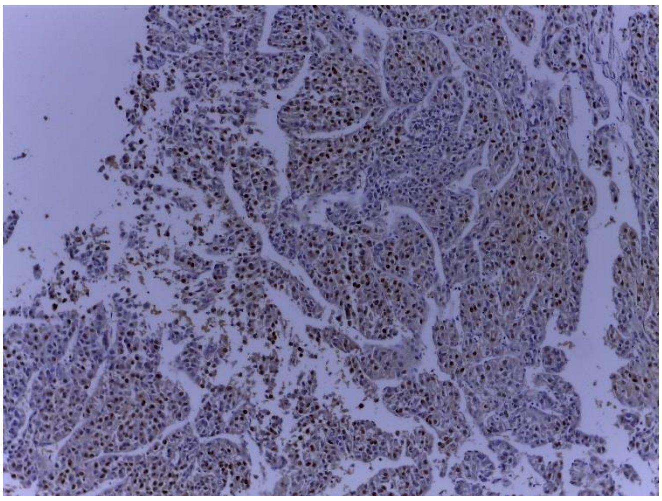

10C

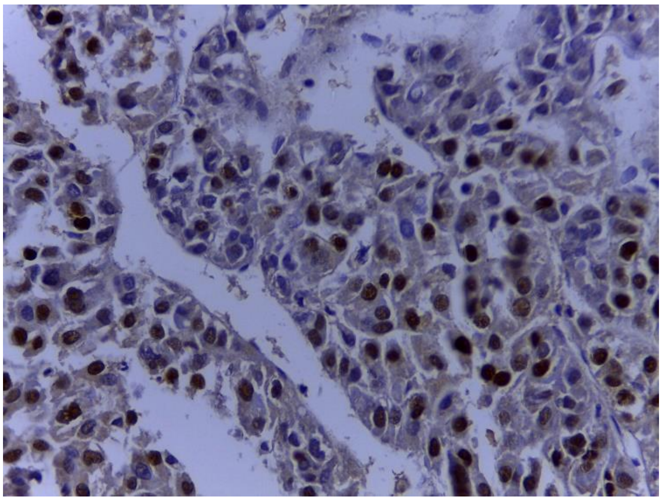

10C-1

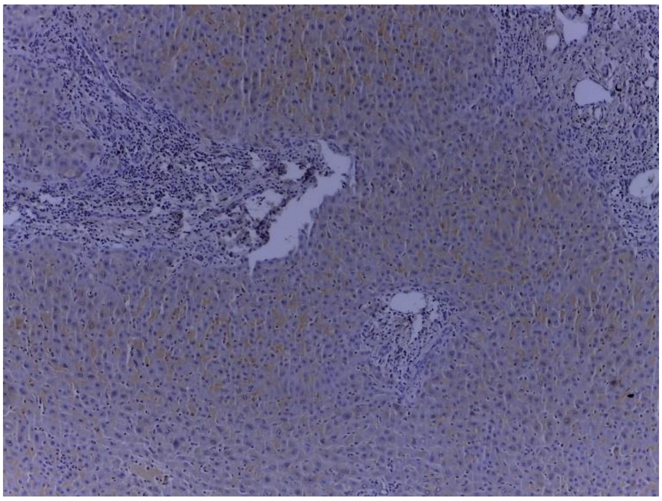

04A

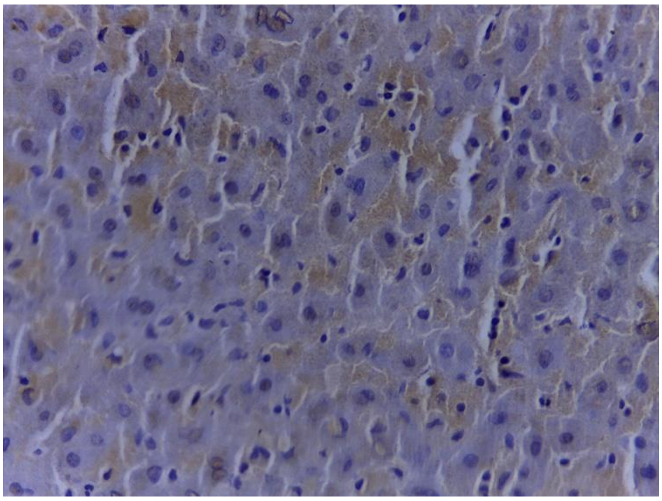

04A-1

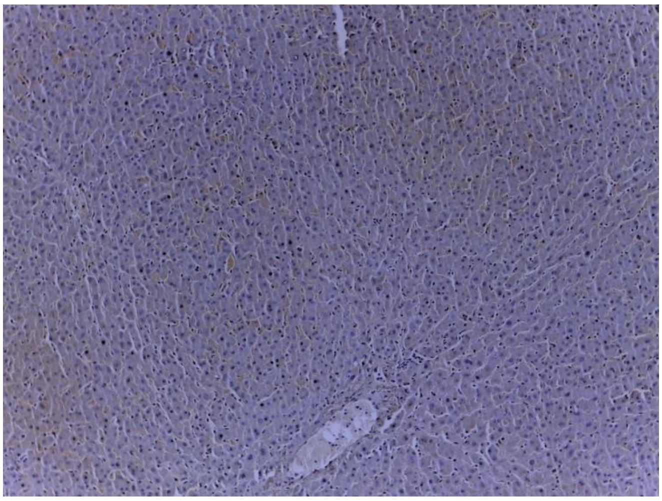

04F

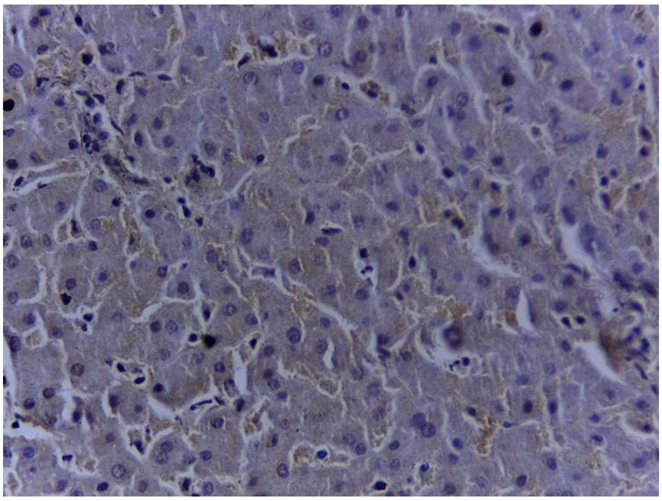

04F-1

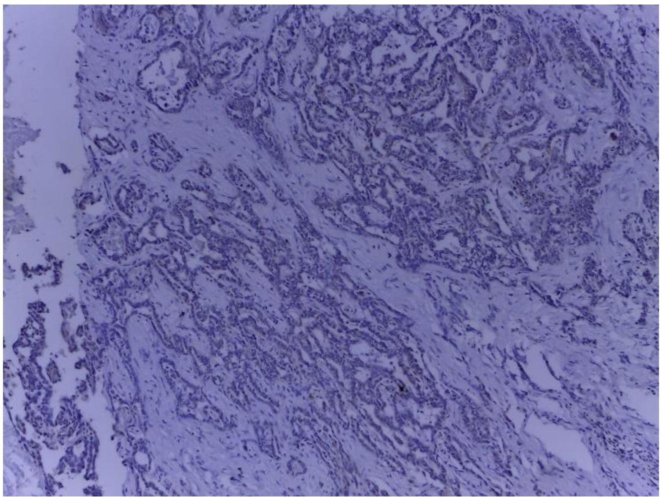

04C

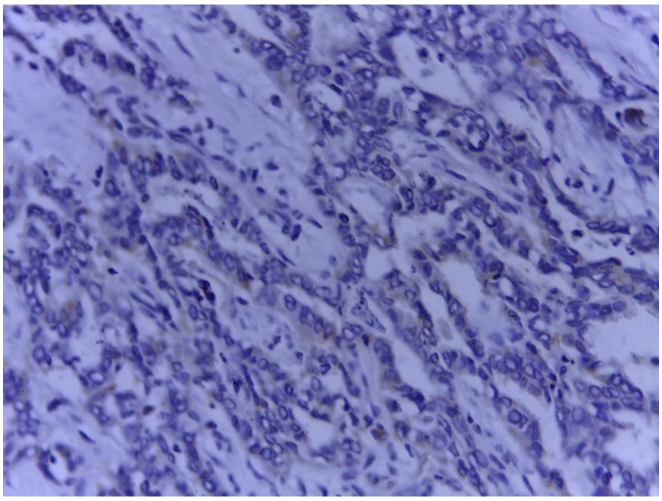

04C-1

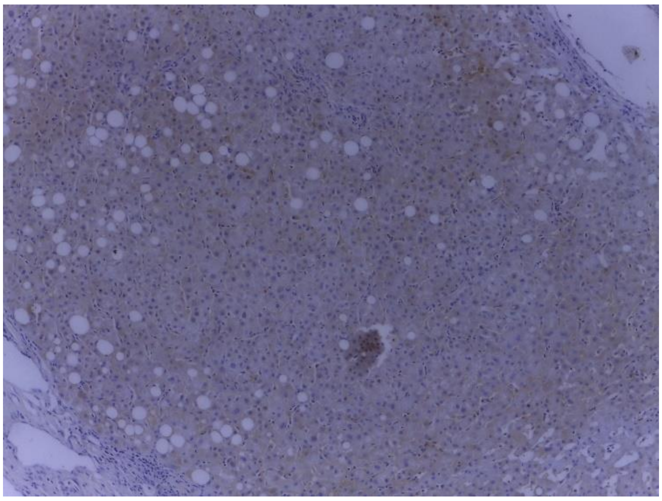

05A

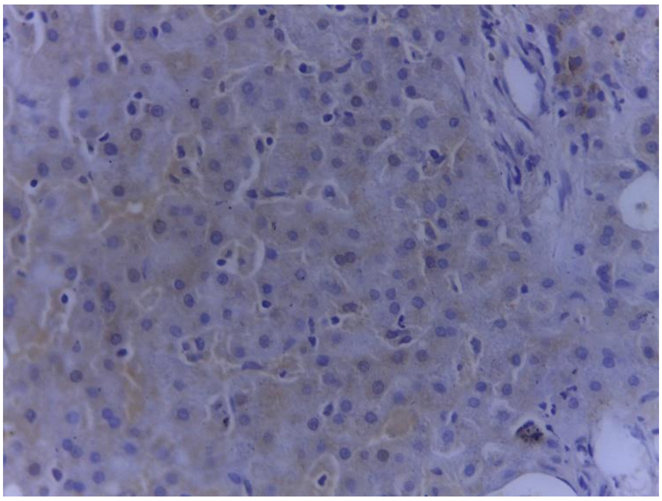

05A-1

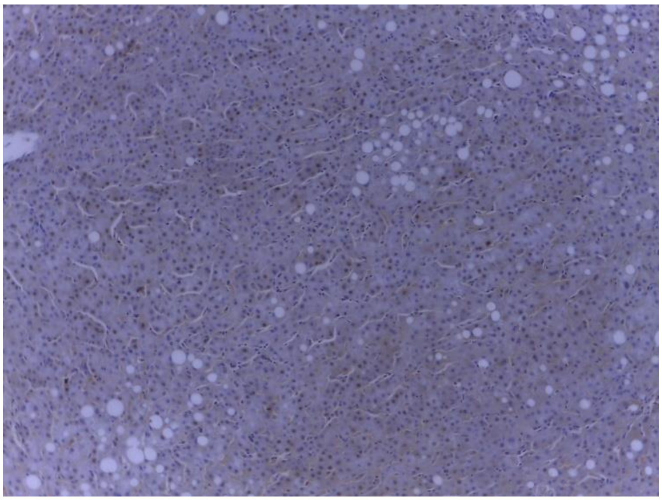

05F

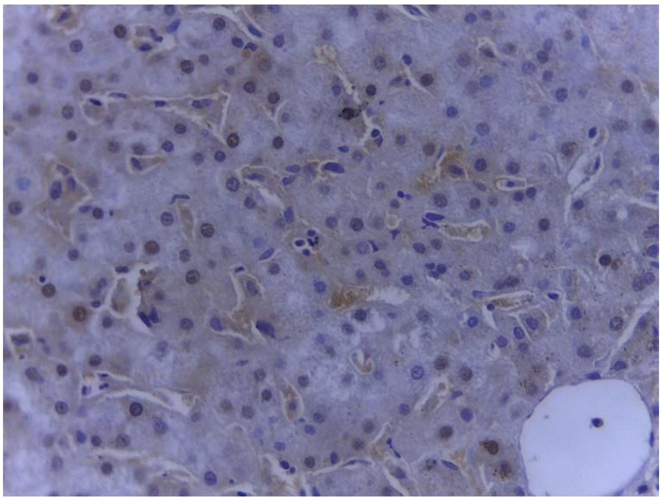

05F-1

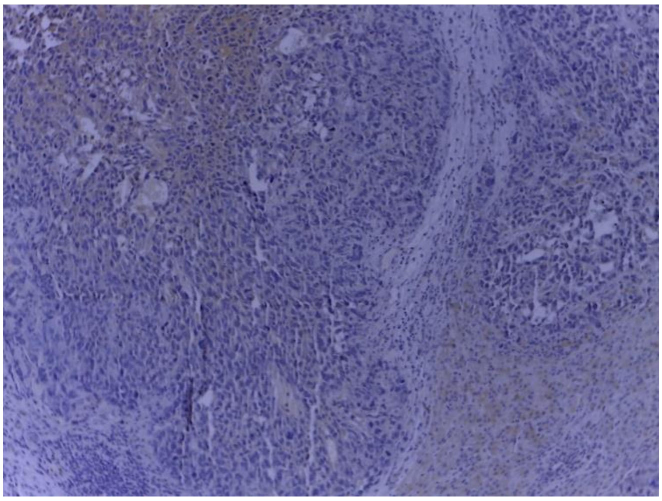

05C

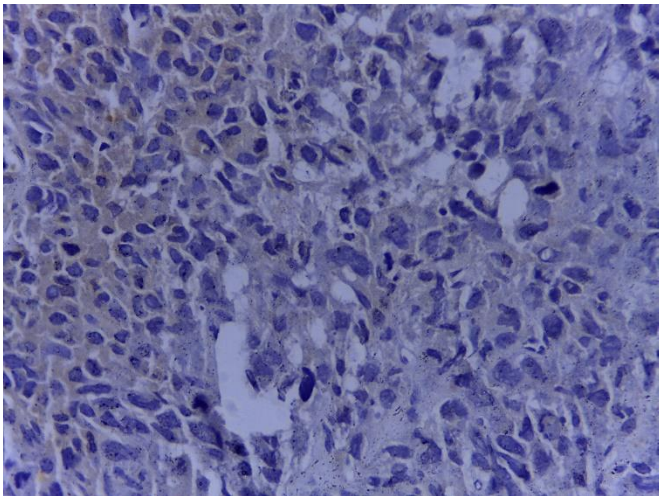

05C-1

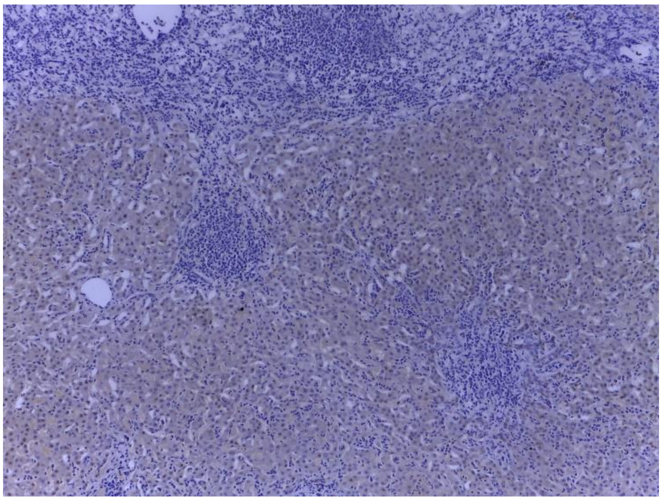

06A

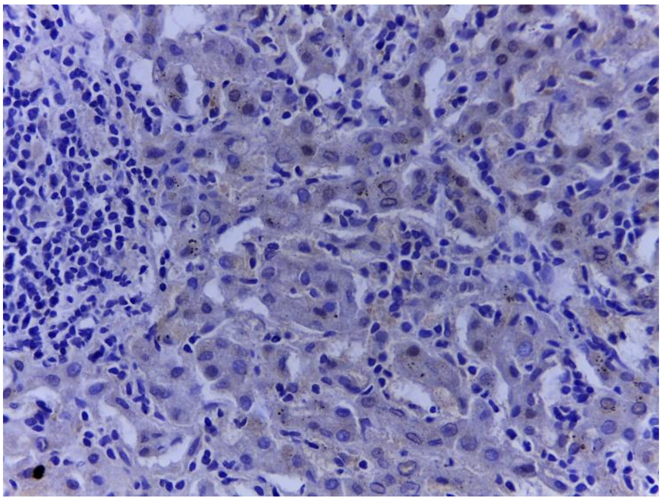

06A-1

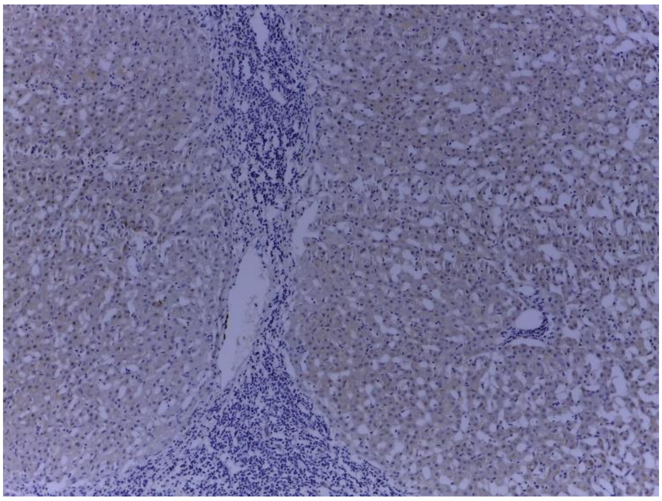

06F

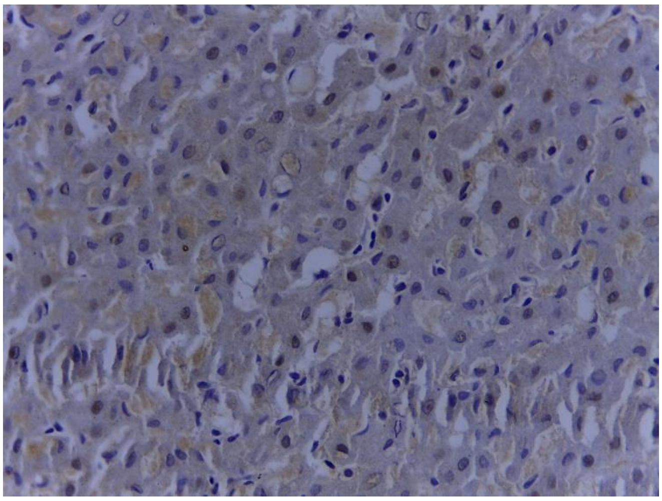

06F-1

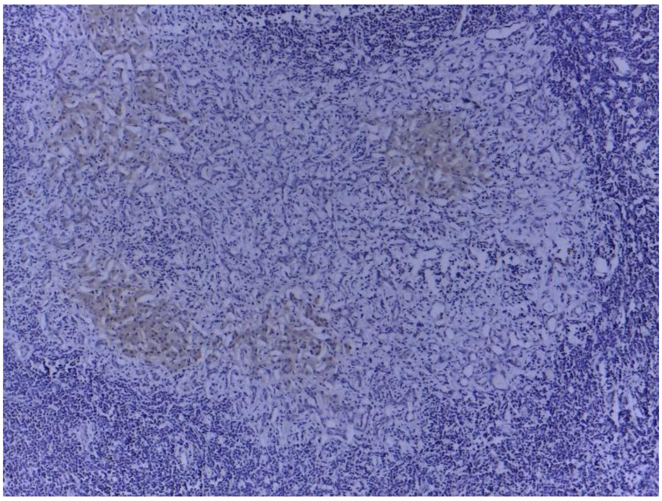

06C

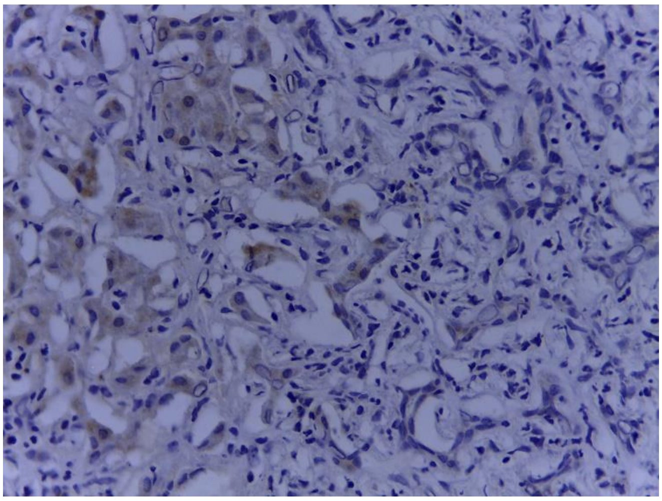

06C-1

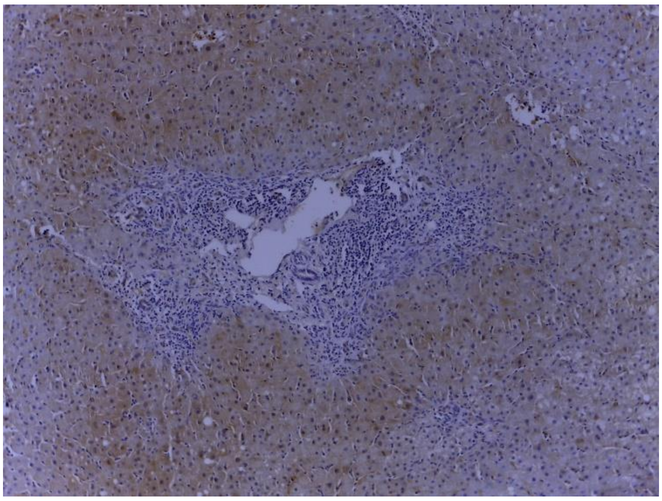

07F

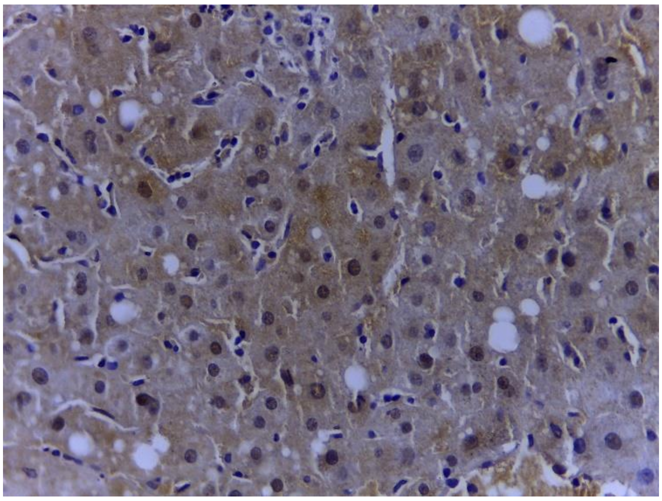
07F-1

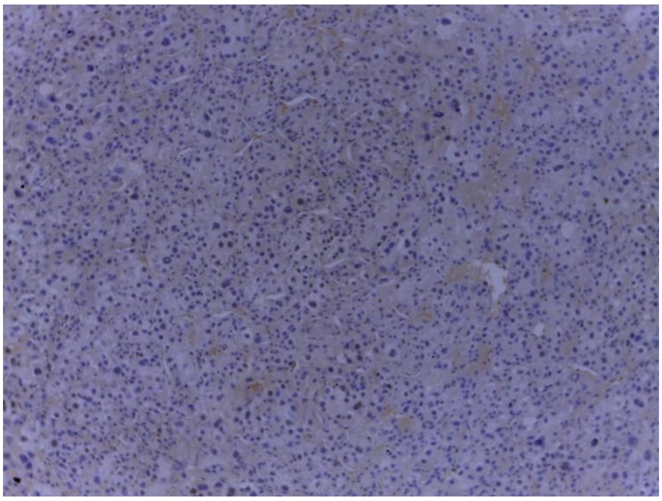
07C

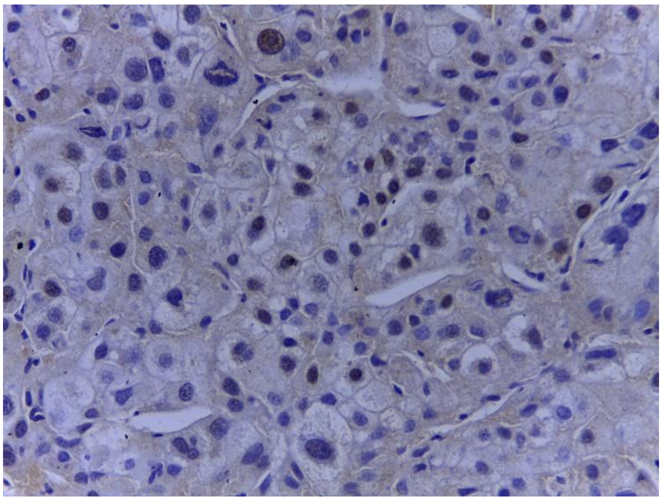
07C-1

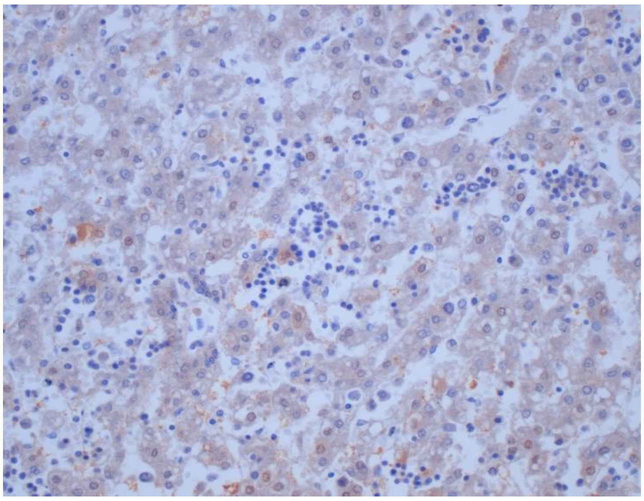
Normal-6

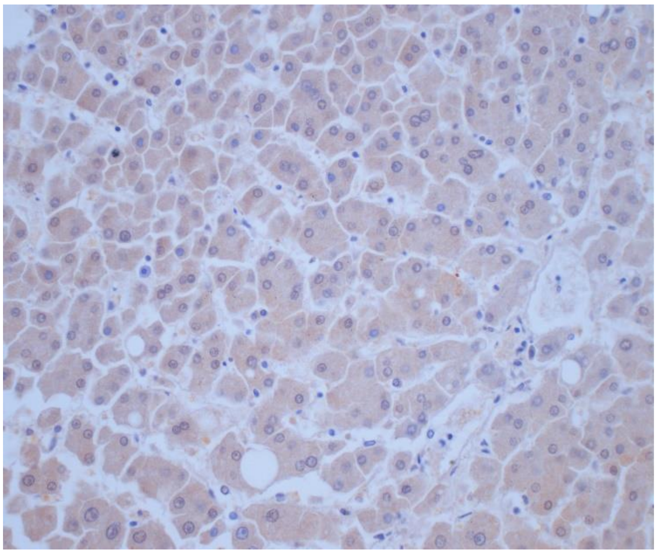
Normal-7

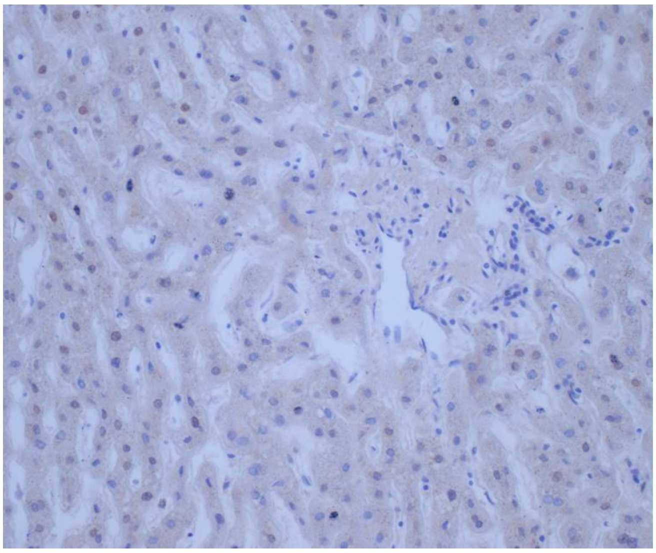
Normal-8

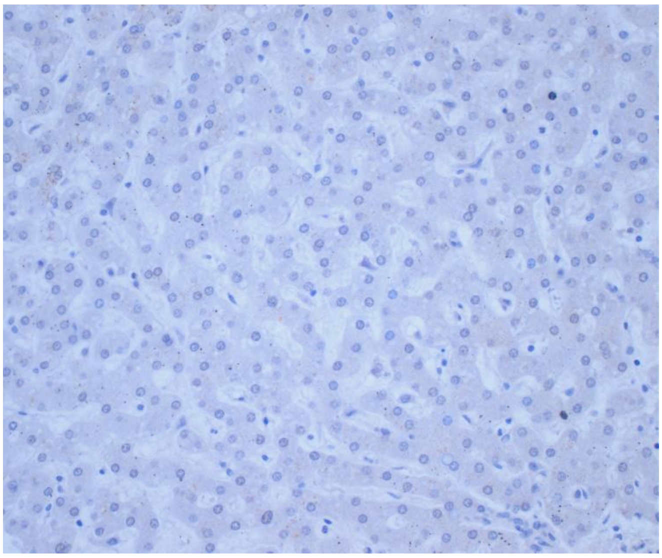

Normal-09

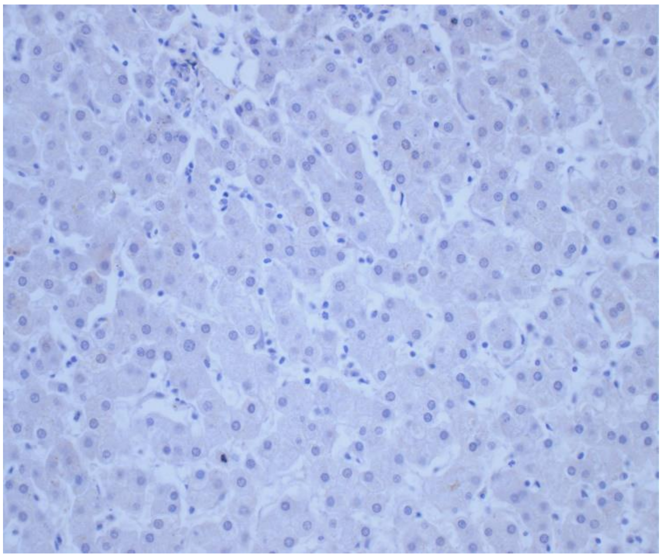

Normal-10

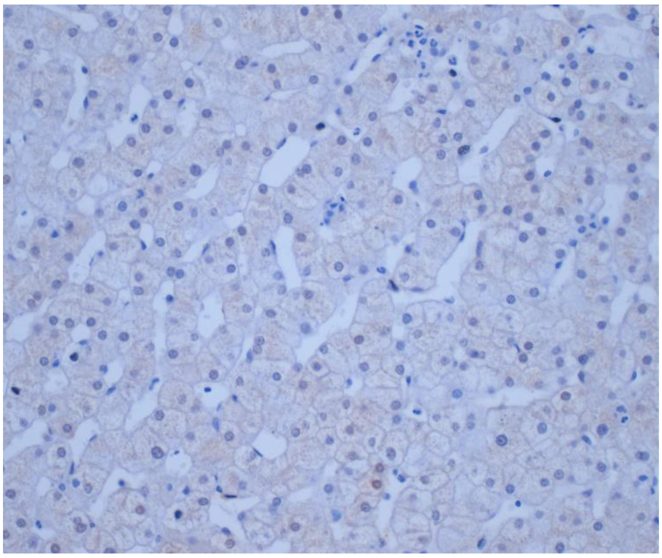

Normal-11

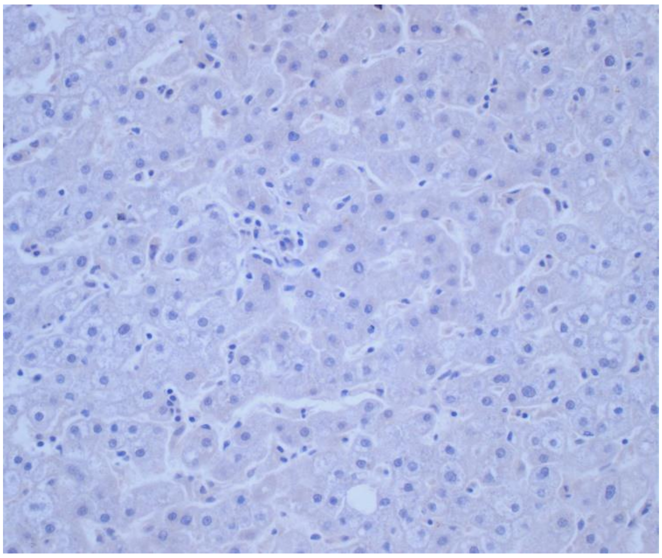

Normal-12

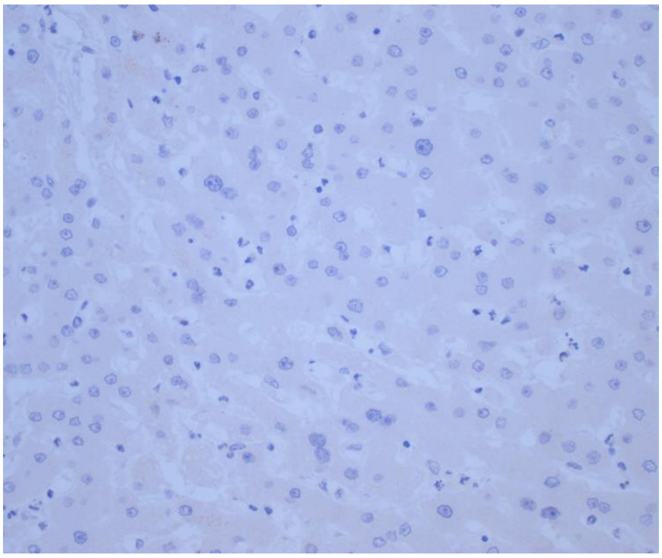

Normal-13

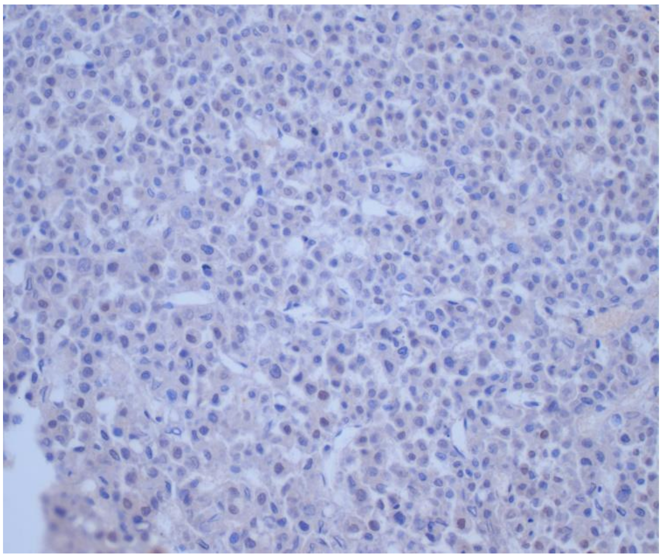

Normal-14

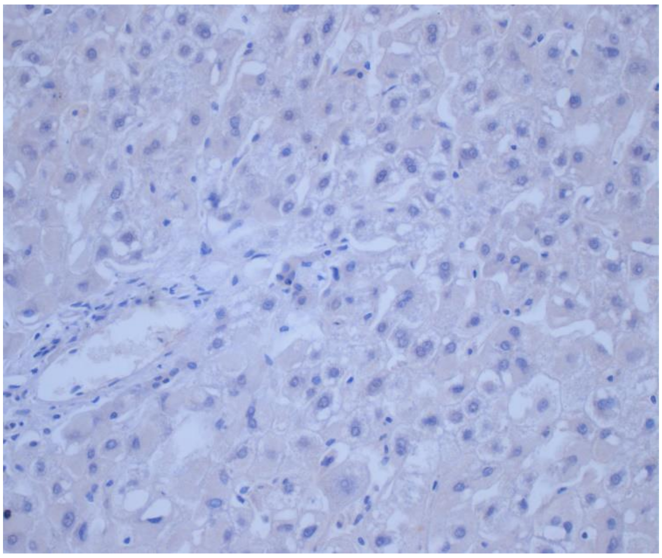

Normal-15

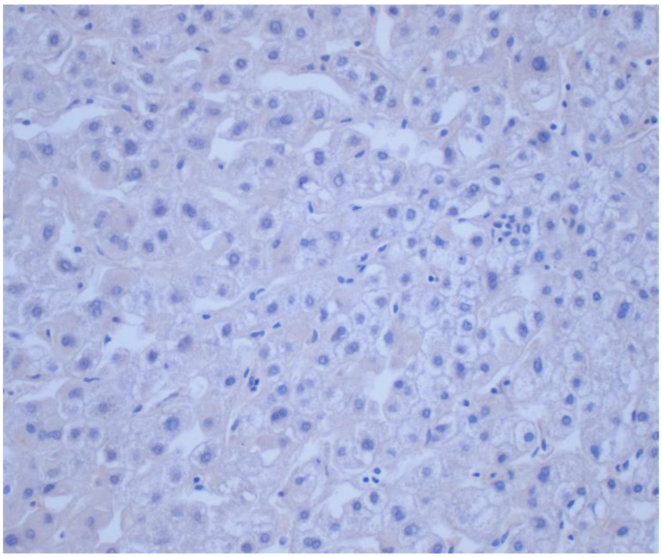

Normal-16

HCV core

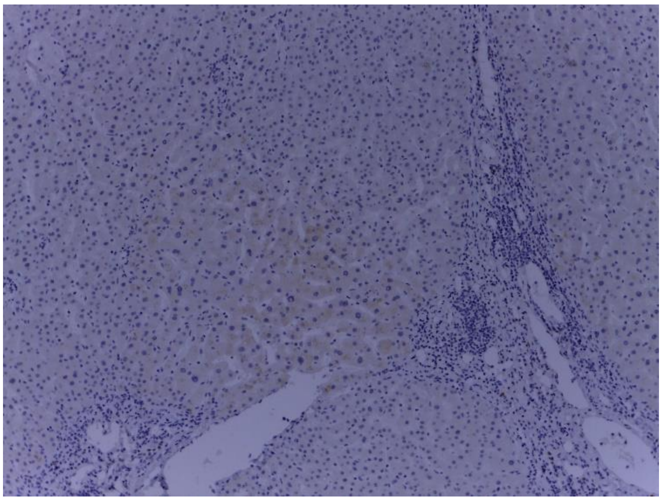

01A

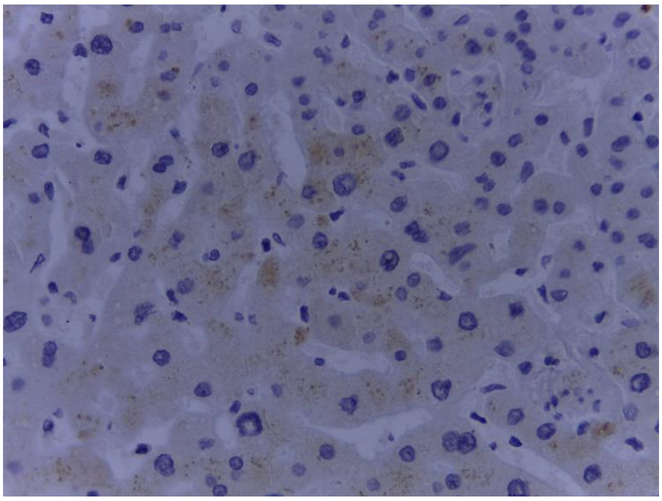

01A-1

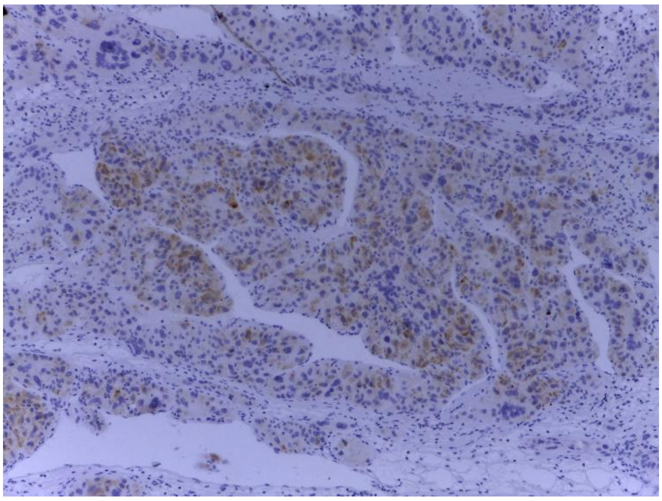

01C

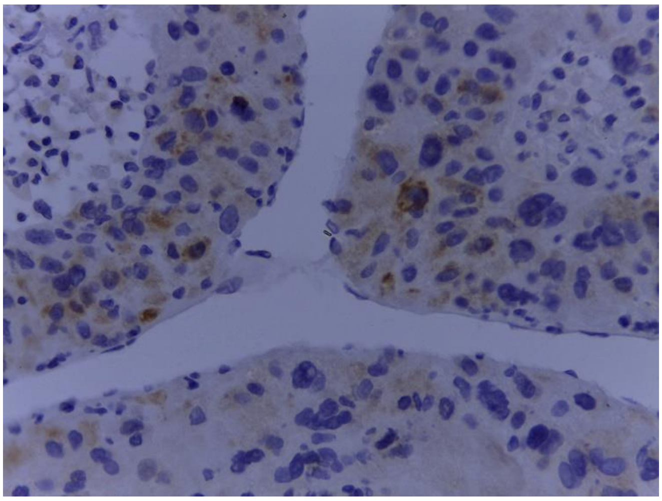

01C-1

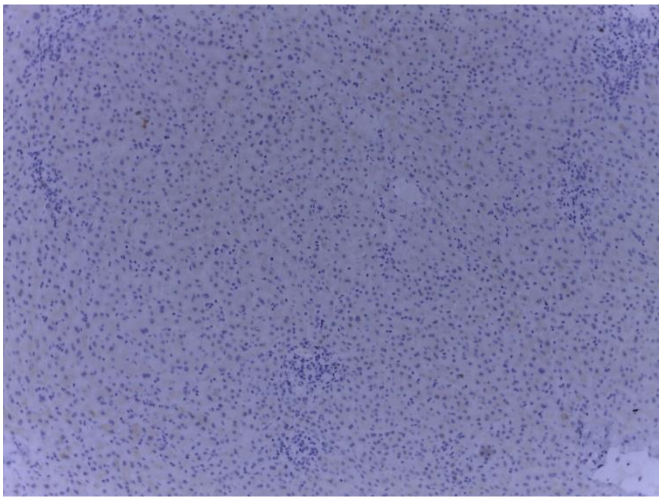

04A

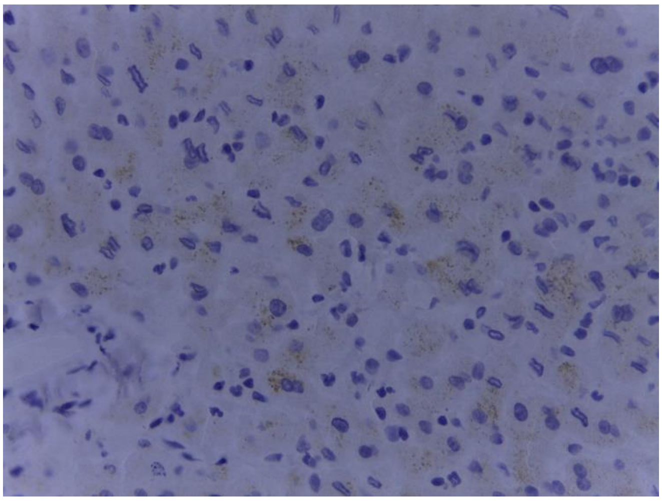

04A-1

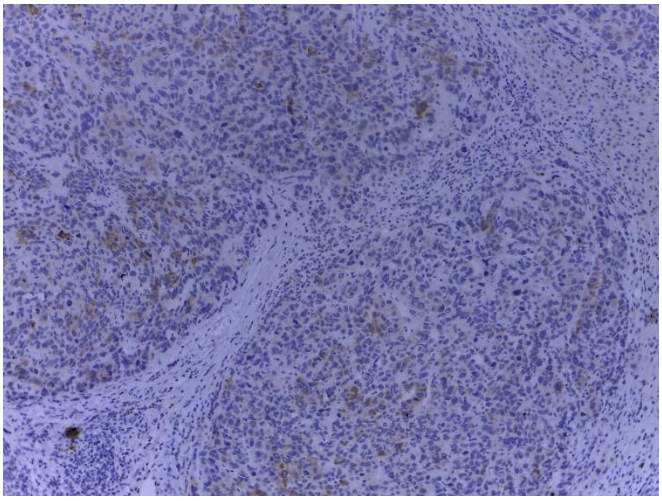
05C

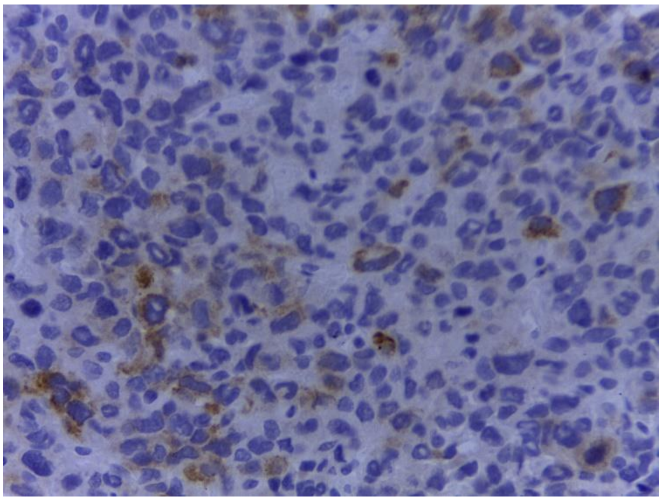
05C-1

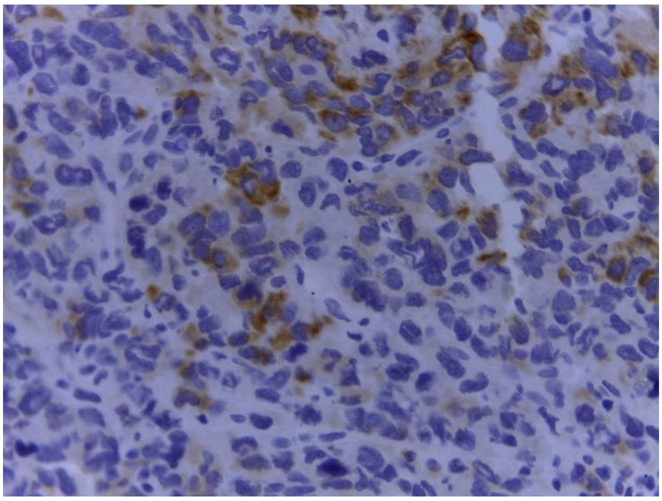
05F

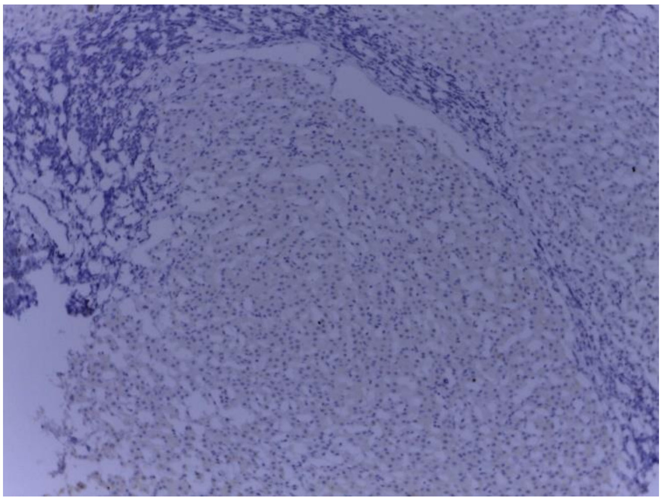
06F

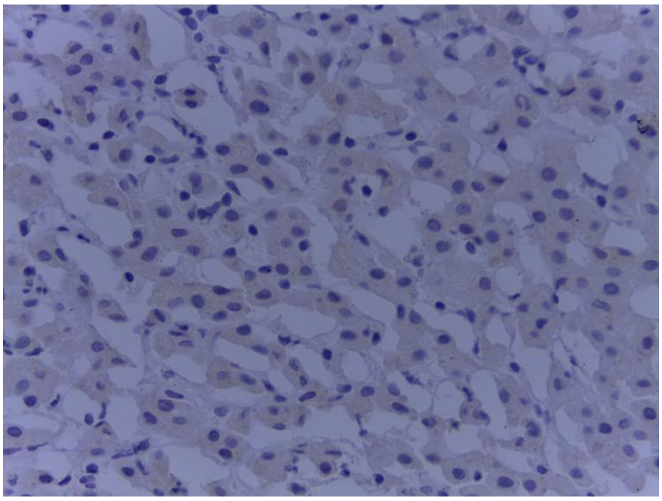
06F-1

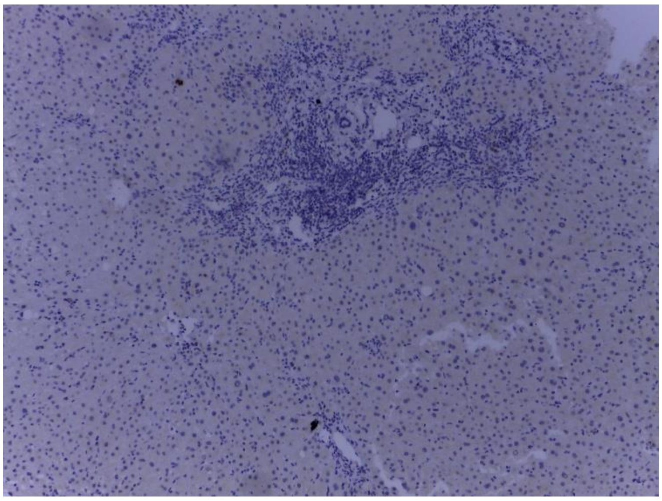
07F

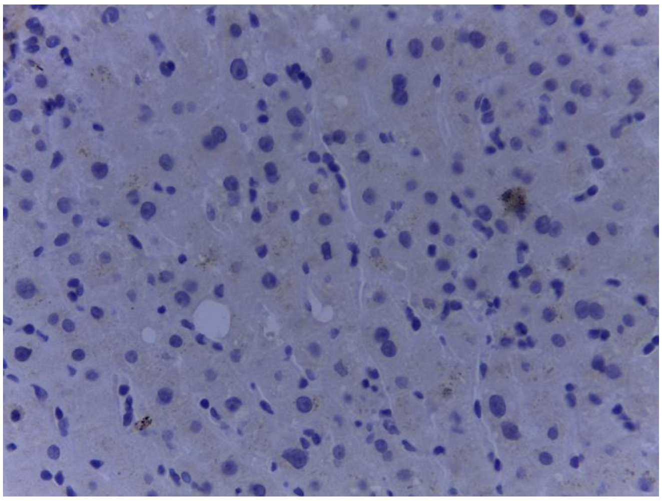

07F-1

HE stained non-HCC liver tissue with HCV infection

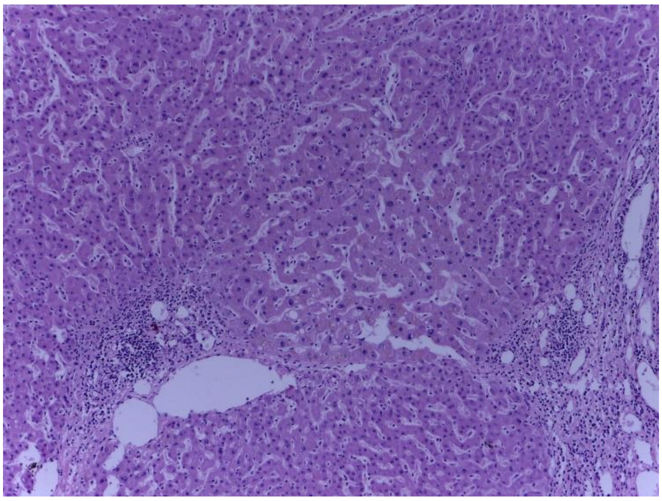

01A

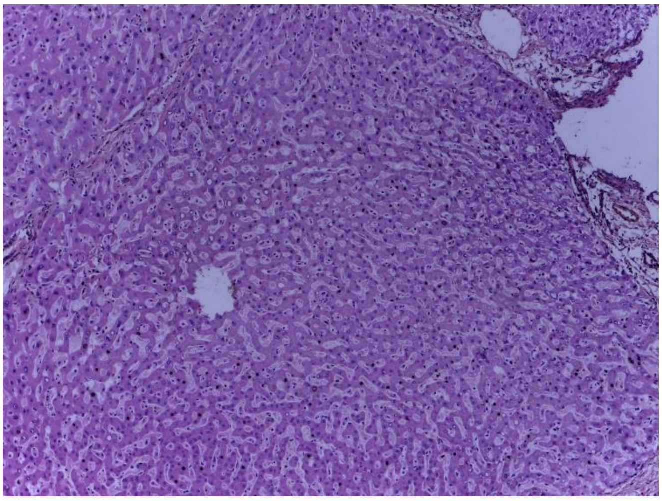

01F

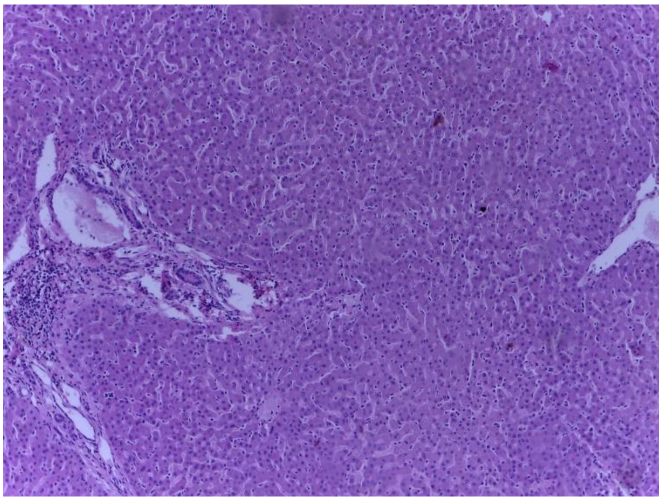

02A

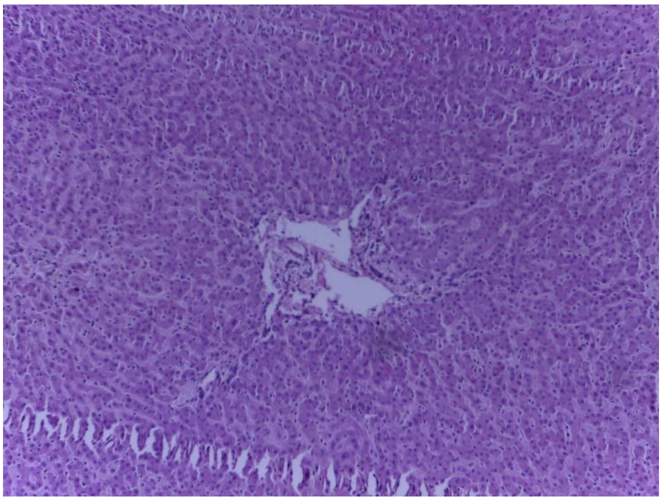

02F

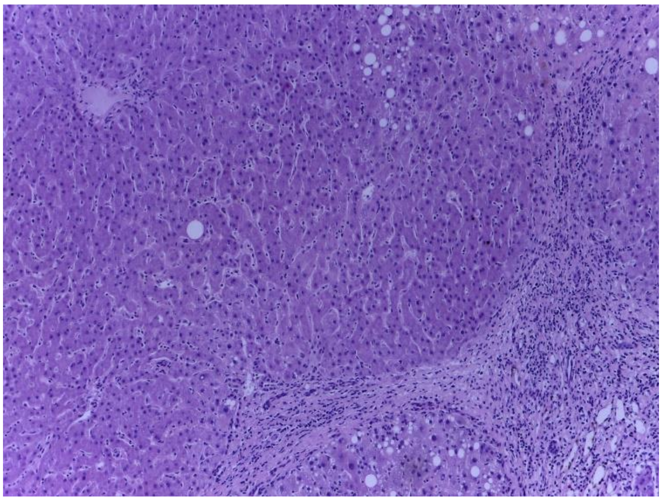

03A

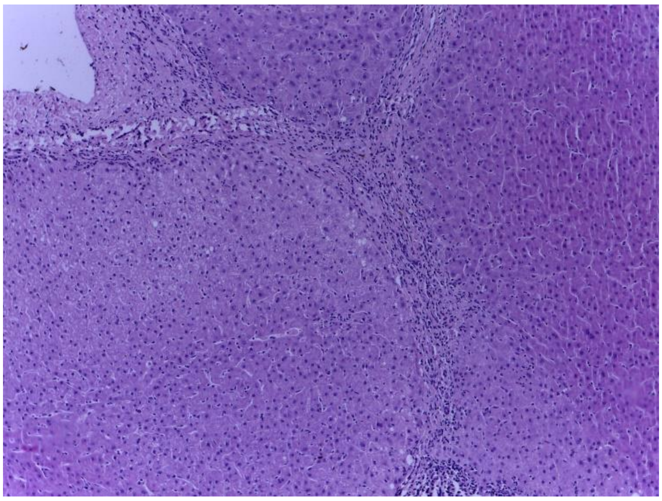

03F

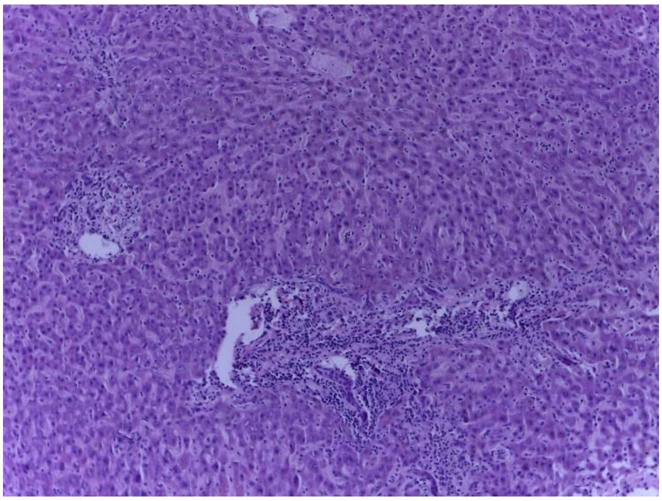

04A

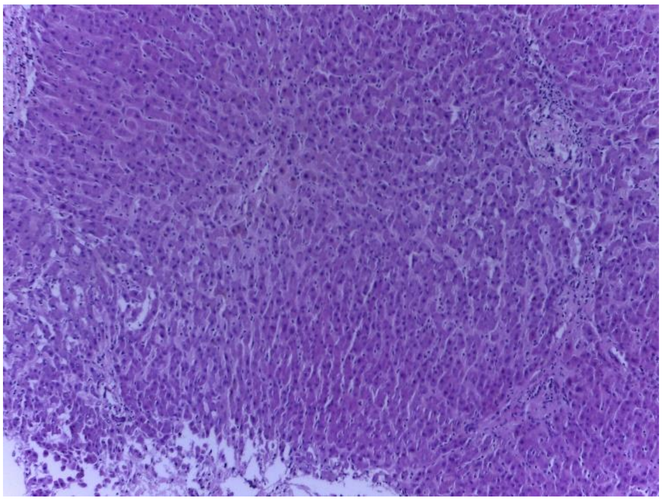

04F

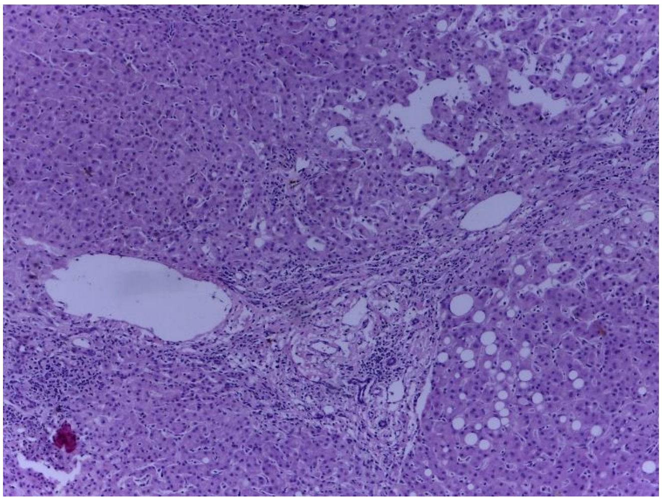

05A

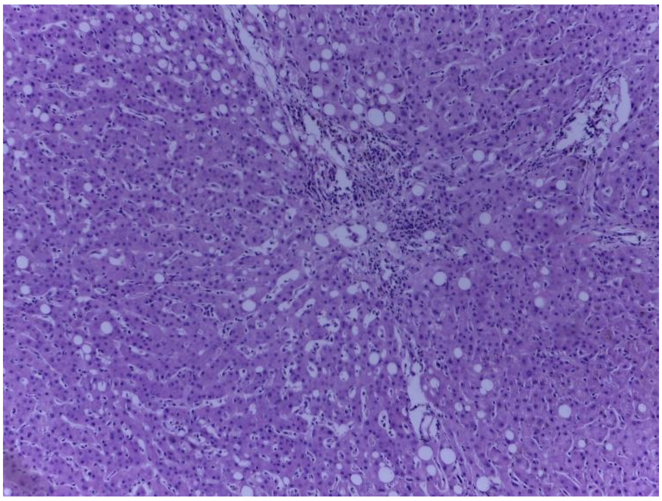

05F

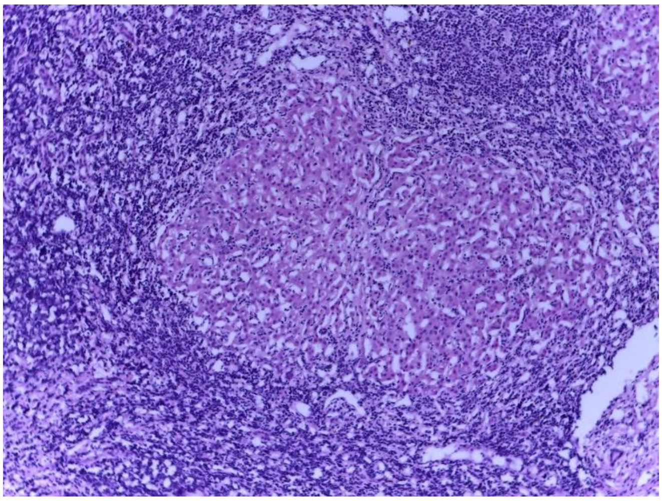

06A

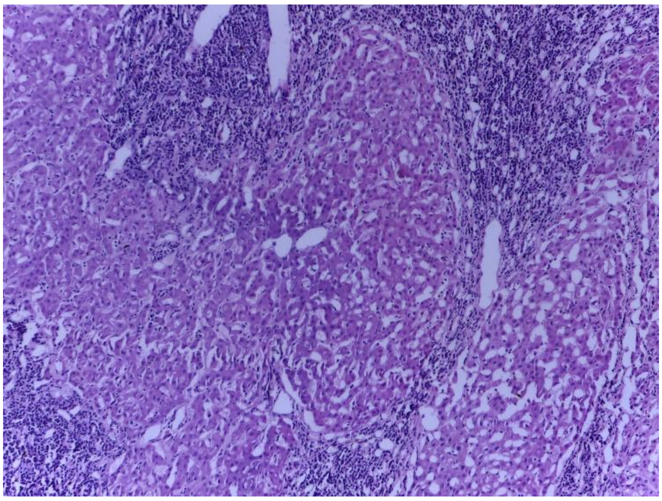

06F

The methylation of CpG island in SOCS-1 gene promoter

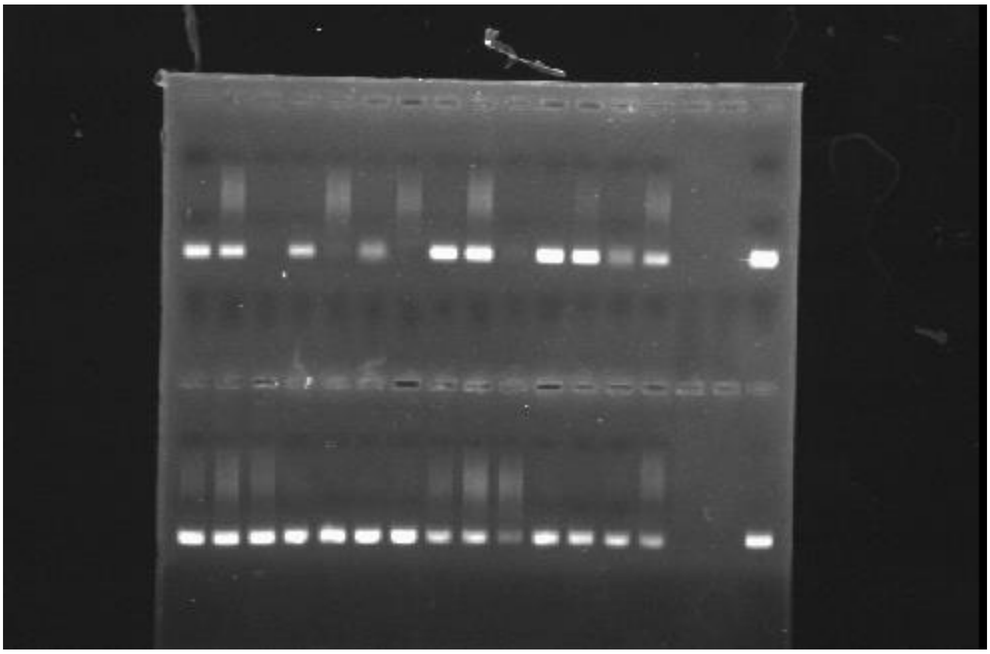

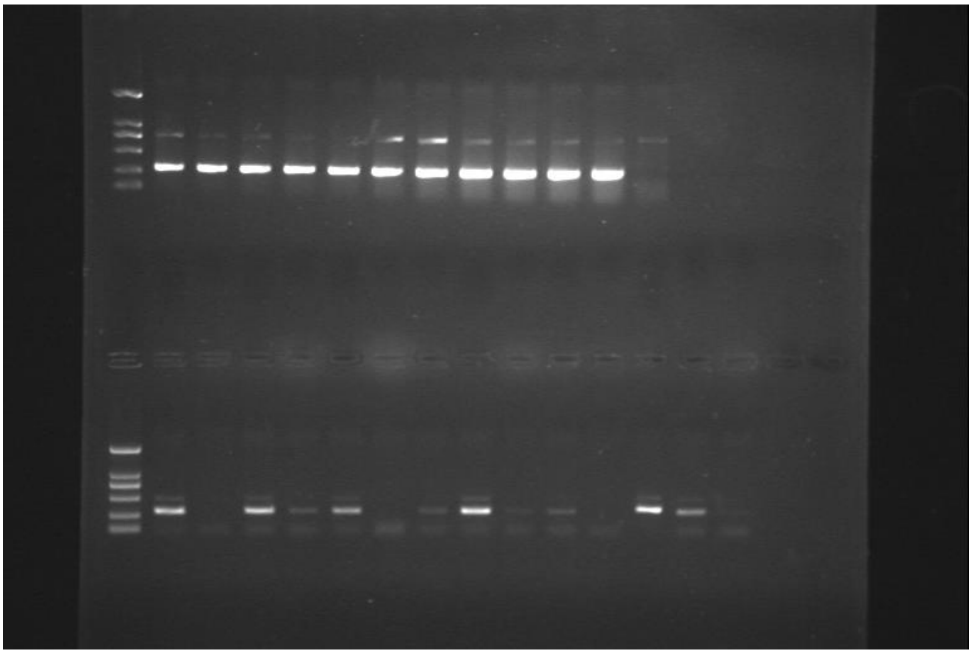

Axiotis Score of SOCS-1 expression in each kind of liver tissues.

A	B	C	D
Normal	HCV-HCC	HCV-Aside	HCV-Far
Y	Y	Y	Y
2	0	2	2
0	4	4	4
1	1	4	4
2	4	1	4
4	0	1	4
6	0	4	1
9	0	6	2
4	2		
0	2		
0			
4			
0			
0			
0			
2			
2			

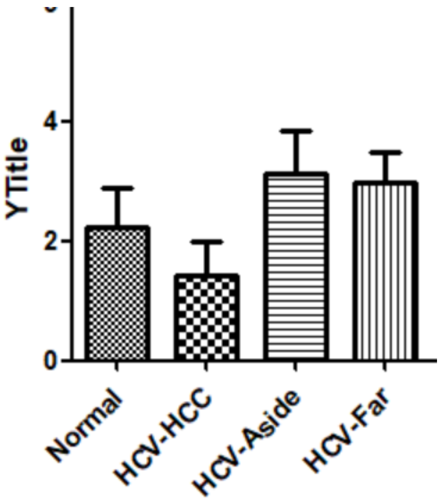

Time course of DAC treatment

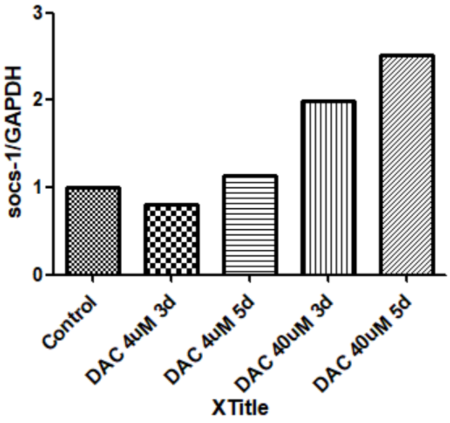

A	B	C	D	E
Control	DAC 4uM 3d	DAC 4uM 5d	DAC 40uM 3d	DAC 40uM 5d
Y	Y	Y	Y	Y
0.995390	0.808508	1.130269	1.986185	2.519842

ICC

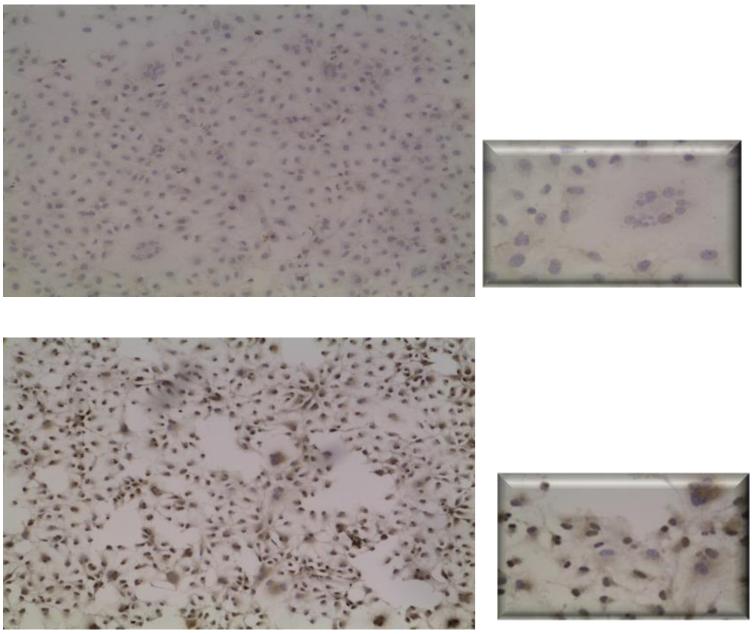

Supplement: Supplementary file 1 [file Data_Sheet_1.PDF]
